# Age-related cortical mechanisms underlying postural control: a systematic review

**DOI:** 10.3389/fnagi.2026.1879821

**Published:** 2026-09-07

**Authors:** Myeongjin Bae, Alexandria Hoang, Katarina Puš, Valerie Nunez, Uros Marusic, Jeannette R. Mahoney

**Affiliations:** 1 Division of Cognitive and Sensorimotor Aging, Department of Neurology, Renaissance School of Medicine, Stony Brook University, Stony Brook, NY, United States; 2 Department of Neurobiology and Behavior, Stony Brook University, Stony Brook, NY, United States; 3 Institute for Kinesiology Research, Science and Research Centre Koper, Koper, Slovenia; 4 Department of Health Sciences, Alma Mater Europaea University, Maribor, Slovenia

**Keywords:** aging, cortical activation, dual-task, mechanical perturbation, postural balance, reactive balance, sensory perturbation, static balance

## Abstract

**Introduction:**

Despite growing interest in age-related cortical mechanisms of postural control, heterogeneity across studies has limited the synthesis of existing evidence. This systematic review aimed to (1) characterize age-related alterations in cortical activity during standing balance tasks with sensory, cognitive, and mechanical challenges; (2) examine the association between cortical activity and postural sway; and (3) characterize the current methodology of assessing cortical activity during standing balance tasks in older adults (OA).

**Methods:**

Relevant research articles were identified through systematic searches of MEDLINE, EMBASE, PsycINFO, and CINAHL from inception to October 8, 2025. Eligible studies measured cortical activity during standing balance under sensory, cognitive, and mechanical challenges using electroencephalography (EEG) or functional near-infrared spectroscopy (fNIRS). We used the Standard Quality Assessment Criteria for quantitative studies for quality assessment.

**Results:**

Thirty-seven studies with a total of 1,302 participants, including 788 OA (60.5%), were included. Most studies (*n* = 29, 78.4%) demonstrated strong methodological quality. Evidence suggested diminished task-dependent cortical modulation and weaker associations between cortical activity and postural sway as task demands increased in OA compared to younger adults (YA). OA tended to show prolonged latencies and reduced amplitudes following mechanical perturbation. Nevertheless, some studies also suggested that OA may still recruit additional cortical resources under specific task conditions, which may reflect compensatory processes.

**Discussion:**

This systematic review indicates that aging may be associated with diminished task-dependent cortical modulation, weaker associations between cortical activity and postural sway, and delayed sensorimotor processing during challenging balance tasks. These findings provide a foundation for future studies aimed at clarifying age-related cortical mechanisms of postural control during standing balance.

**Systematic review registration:**

https://www.crd.york.ac.uk/PROSPERO/, identifier CRD420251159695.

## Introduction

Falls are common among older adults (OA), with approximately 30% experiencing a fall each year (Ganz and Latham, 2020). Falls prevalence increases with age, with about 50% of OA over the age of 80 experiencing a fall annually (Ambrose et al., 2013; Ganz and Latham, 2020). Falls are associated with increased activities of daily living impairment, restricted social participation, and lower quality of life in OA (Schoene et al., 2019; Adam et al., 2024). Among various fall risk factors, such as declines in balance, gait, sensory function, and cognitive function, deteriorated balance is a primary risk factor (Ambrose et al., 2013; Ganz and Latham, 2020). Therefore, understanding the underlying neural mechanisms of postural control is critical for effectively reducing fall risk in OA.

Balance/postural control is defined as the ability to maintain, achieve, or restore balance during any posture or activity (Pollock et al., 2000), resulting from the complex integration of motor, sensory, and cognitive components (Horak, 2006). Balance function declines with age (Degani et al., 2017), and this decline becomes more pronounced when sensory inputs are unavailable, attenuated, or perturbed. For example, compared to a comfortable stance with eyes open, OA demonstrate greater postural instability when their visual (e.g., eyes closed, optical flow), somatosensory (e.g., foam surface), vestibular, or multisensory modalities are perturbed (Lin et al., 2021; Hu et al., 2023). Similarly, performing balance and cognitive tasks simultaneously (i.e., dual-tasking) or under mechanically challenging conditions (e.g., moving balance platform) has also been shown to demonstrate larger postural sway in OA as compared to younger adults (YA) (Petrigna et al., 2021; Hu et al., 2023). However, despite well-established evidence of decreased balance function primarily assessed using kinematic measurements, much less is known about age-related cortical mechanisms of postural control, especially when combined with varying sensory, cognitive, and mechanical challenges.

The brain plays a crucial role in maintaining balance (Jacobs and Horak, 2007), and the level of cortical involvement and higher-order cognitive processes needed for adequate postural control increases with age (Wittenberg et al., 2017; Li et al., 2018), largely attributable to declines in sensory or multisensory function (Mahoney et al., 2011; Mahoney et al., 2014), structural and functional brain changes associated with aging (Seidler et al., 2010), and increased demand of attentional resources (Holtzer et al., 2011). Advancements in neuroimaging techniques, such as electroencephalography (EEG) and functional near-infrared spectroscopy (fNIRS), enable researchers to assess direct, real-time cortical involvement during balance tasks. Prior studies using these approaches have examined multiple dimensions of cortical activity to investigate altered cortical responses during postural control in OA, including the power spectrum of frequency bands, functional connectivity, event-related potentials (ERPs), and hemodynamic responses, particularly under various sensory, cognitive, and mechanical challenges (Lin et al., 2021; Palmer et al., 2021; Kahya et al., 2022; Lehmann et al., 2022; Chen et al., 2024).

Among the various dimensions of cortical activity, power spectra in the delta (0.5–4 Hz), theta (4–7 Hz), alpha and mu (8–12 Hz), beta (13–30 Hz), and gamma (> 30 Hz) bands measured using EEG have been widely used to investigate the cortical mechanisms underlying balance, as each frequency band is associated with distinct motor and cognitive processes. For example, compared with single-task conditions, OA exhibited increased delta and theta power in frontal and frontocentral regions during dual-task conditions, with delta power associated with greater postural demands and theta power associated with higher cognitive task difficulty (Ozdemir et al., 2016). Alpha power plays a key role in sensory processing and inhibitory control, with increased alpha power reflecting functional inhibition and decreased alpha power reflecting heightened motor and cortical activation (Klimesch, 2012; Hülsdünker et al., 2015). In particular, alpha-band activity over sensorimotor regions, commonly referred to as mu power, is closely associated with motor and somatosensory processing, where mu power decreases during movement preparation and execution and increased postural demands in OA (Malcolm et al., 2021). Beta-band activity appears to support postural control in a context-dependent manner. Decreased beta power may reflect flexible sensorimotor processing, whereas increased beta power may indicate greater cognitive effort and sensory reweighting under challenging conditions (Seeber et al., 2014; Tsai et al., 2022; Huang and Ferris, 2023). Gamma-band activity is associated with higher-order cognitive processing and sensorimotor integration, with increased gamma power during challenging mechanical perturbations reflecting greater cortical recruitment to maintain standing under demanding conditions (Ozdemir et al., 2018). However, methodologies in previous studies have varied in terms of cortical measures, regions of interest (RoI), and balance paradigms, leading to inconsistent conclusions and limiting a comprehensive understanding of age-related cortical mechanisms during balance.

To our knowledge, a recent scoping review investigated EEG power spectrum correlates of standing or walking in OA (Kahya et al., 2026). However, its scope was limited to EEG spectral measures and, as a scoping review, it did not provide a comprehensive synthesis of other cortical activity outcomes relevant to standing balance, including functional connectivity, event-related potentials, and hemodynamic responses. Therefore, the main objective of this systematic review was to investigate age-related differences in the neural mechanisms underlying standing balance across a broad range of measures, including spectral power, functional connectivity, event-related potentials, and oxygenated hemoglobin responses, by synthesizing studies using EEG and fNIRS. Specifically, this review aims to: (1) characterize age-related alterations in cortical activity during standing balance tasks with sensory, cognitive, and mechanical challenges; (2) examine the association between cortical activity and postural sway; and (3) characterize the current methodology of assessing cortical activity during standing balance tasks in OA.

## Methods

This systematic review adhered to the Preferred Reporting Items for Systematic Reviews and Meta-Analyses (PRISMA) guidelines (Page et al., 2021), and the review protocol was registered on the international database of prospectively registered systematic reviews in health and social care [International Prospective Register of Systematic Reviews (PROSPERO); Registration number: CRD420251159695].

### Information sources and study selection

We performed a comprehensive systematic search using MEDLINE, Embase, PsycINFO, and CINAHL from inception to October 8, 2025. A combination of database-specific MeSH terms and keywords was used to develop the search query. Briefly, the query included keywords such as (“aging” OR “older adult*”) AND (“balance” OR “postur*”) AND (“fNIRS” OR “EEG” OR “neural activit*”). The full search query used in each database is provided in Supplementary Table 1. Additionally, the authors (M.B. and A.H.) performed website searching (e.g., Google Scholar) and manually checked references cited within included studies to further identify potential articles of interest (i.e., citation search method).

The literature screening was performed by two authors (M.B. and A.H.) independently using the Covidence web-based collaboration software for systematic review (Veritas Health Innovation, Australia). Covidence software removes duplicated literature automatically first, which then enables authors to efficiently perform title and abstract screening. Full-text screening was conducted using the study’s Population, Intervention, Comparison, Outcome, and Study design (PICOS) criteria (see below). Discrepancies in article inclusion or exclusion were discussed and resolved by the authors after completing the abstract and full-text screening, respectively.

### PICOS eligibility criteria

Healthy, non-demented adults aged 60 or above, including those with mild cognitive impairment, were included in this systematic review. However, studies were excluded if they involved OA with neurodegenerative diseases [e.g., Parkinson’s disease, Amyotrophic Lateral Sclerosis (ALS), Alzheimer’s Disease, multiple sclerosis, etc.] or musculoskeletal conditions (e.g., osteoarthritis), even if healthy OA were included as a comparison group. The intervention criterion was not applicable in this review. Regarding the comparison criteria, this review did include studies that involved healthy comparison groups [e.g., healthy young (YA) or middle-aged adults (MA)]. Studies were eligible if they used EEG or fNIRS to measure cortical activity during standing postural control tasks combined with sensory manipulation (e.g., narrow base of support, visual interference, foam surface), cognitive (e.g., motor-motor, cognitive-motor), and mechanical challenges (e.g., externally induced platform translations). Observational studies published in English were included in this review, such as cross-sectional design, baseline data of clinical trials, secondary data analysis, and prospective cohort design. Protocol papers, conference abstracts, and case reports were excluded.

### Quality assessment of included studies

Two authors (M.B. and K.P.) independently performed the methodological quality assessment using the Standard Quality Assessment Criteria for quantitative studies (Kmet et al., 2004). Discrepancies of evaluation between the authors were discussed and resolved until reaching consensus. The quality assessment tool has 14 items that assess the range of the study’s methodological quality, including subject selection/characteristics, robustness of measurement, and detailed result reporting. Each item was answered with Yes (2 points), Partial (1 point), No (0 points), and N/A (not applicable). The final scores were calculated using the following procedure: (1) Total sum = (number of “Yes” × 2) + (number of “Partial” × 1); (2) Total possible sum = 28 - (number of “N/A” × 2); and (3) Final score = Total sum/Total possible sum. Following the approach used in previously published systematic reviews, higher final scores reflected greater methodological quality, and final scores were categorized as low (< 0.70), good (0.70–0.79), or strong (≥ 0.80) (Lindsay and Thiyagarajah, 2021; Abou et al., 2022; Bae and VanNostrand, 2025).

### Data extraction

The initial extraction of data from the included studies was completed by one author (M.B.), and then its accuracy was subsequently reviewed by another author (K.P.). The extracted data included: (1) study characteristics: study design, country, (2) participants’ characteristics: sample size, age, sex, comparison group, (3) balance protocol: balance tasks, balance measures, and behavioral findings, (4) cortical activity measures, (5) RoI and/or spatial location, (6) cortical activity findings, and (7) associations between cortical activity and postural sway.

### Data synthesis

The characteristics of the studies included in this systematic review were synthesized into three main categories: (1) demographic characteristics, (2) study methodology, and (3) main findings. The demographic characteristics summarize the sample size, age, sex, and comparison group reported in the included studies. Regarding study methodology, we describe the balance tasks, balance measures, brain measures, and ROIs. The main findings of this review were characterized as age-related (e.g., OA vs. YA) and task-related (e.g., baseline vs. challenging tasks) behavioral and neural patterns in OA during standing balance tasks involving sensory, cognitive, and mechanical challenges. Furthermore, we determined how cortical activity was associated with balance performance in OA. Given that all fNIRS studies included in this review reported oxygenated hemoglobin (HbO_2_), a robust and reliable measure that is more closely correlated with neural activity compared to deoxygenated hemoglobin (HHb) (Strangman et al., 2002; Plichta et al., 2007; Herold et al., 2017), our cortical activation results were based on HbO_2_. For EEG studies, the primary outcomes in this review included changes in spectral power within the delta, theta, alpha and mu, beta, and gamma bands during standing balance tasks. Various functional connectivity, event-related potentials, and other EEG-derived measures, such as phase-amplitude coupling (PAC), were synthesized as secondary outcomes when available. The PAC, an index of cortico-posture coupling, quantifies the extent to which the amplitude of band-specific EEG oscillations varies according to the phase of postural fluctuations. Stronger PAC reflects greater temporal coordination between cortical oscillations and postural dynamics during postural control (Chen et al., 2021; Chen et al., 2024).

## Results

### Literature search and extracted studies

**FIGURE 1:**
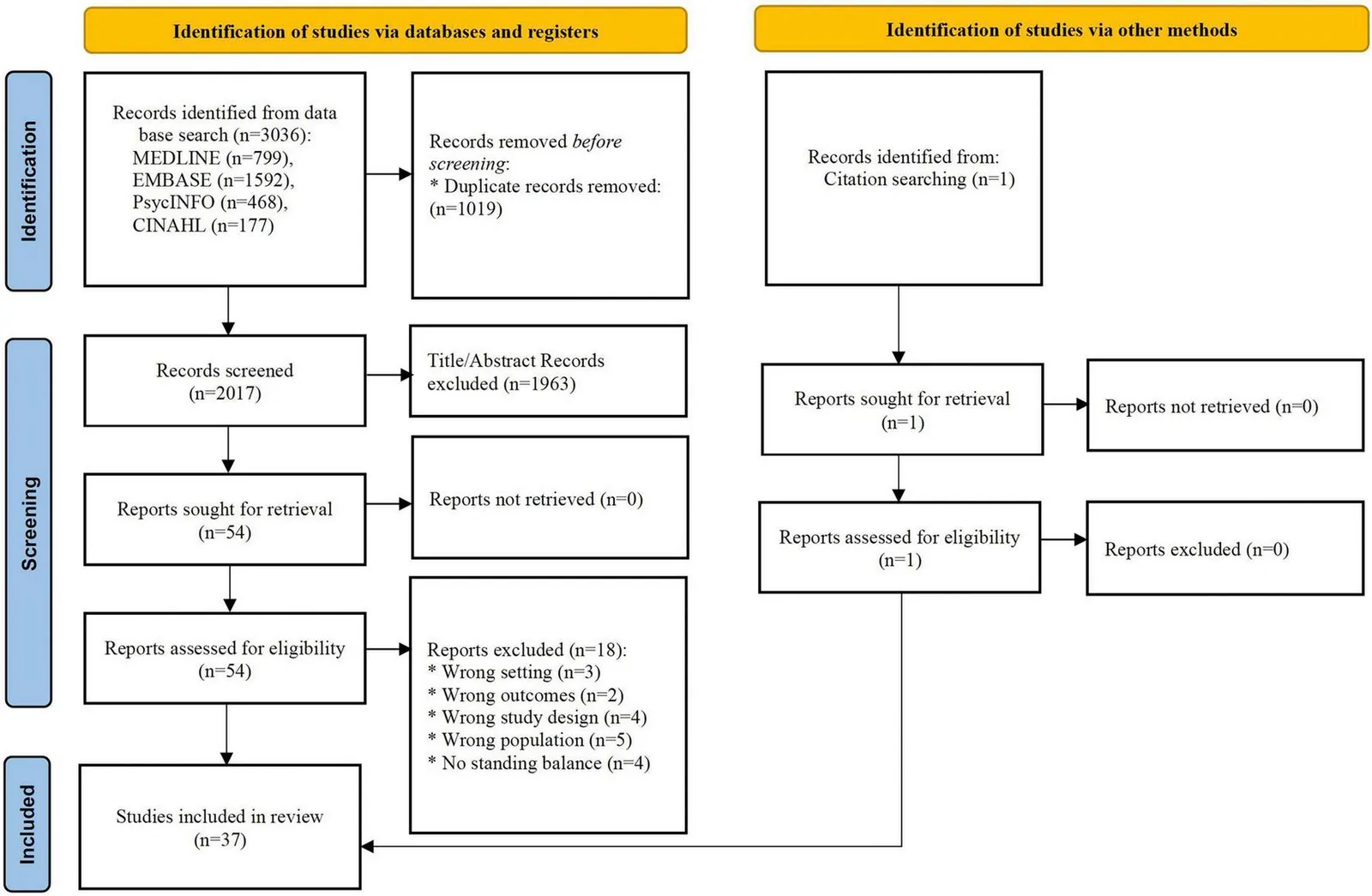
PRISMA flow diagram.

The initial literature search from the above-reported databases produced 3036 articles, of which 1019 duplicates were automatically excluded by the Covidence software. Of the 2017 remaining studies, abstract/title screening removed 1963 articles, yielding a total of 54 studies for full-text review. After excluding studies (*n* = 18) that did not meet our PICOS criteria through full-text screening, a total of 36 studies were identified to be included in this systematic review. One additional article identified from the citation searching met eligibility criteria, resulting in a grand total of 37 studies included in this systematic review. The detailed PRISMA flowchart is provided in Figure 1.

### Quality assessment

Of the 37 studies, 29 (78.4%) demonstrated strong methodological quality, 7 (18.9%) demonstrated good methodological quality, and 1 (2.7%) demonstrated low methodological quality. Most studies (*n* = 34) did not meet or partially met the criterion of “*Controlled for confounding?*” Only a few studies (*n* = 9) conducted a power analysis for sample size calculation and recruited an appropriate number of participants. Several studies did not properly report variance estimates for their main results (*n* = 13), nor did they state a clear participant recruitment strategy (*n* = 17). Quality score of each study is shown in Table 1, and details for each criterion are provided in Supplementary Table 2.

**TABLE 1:** Characteristics of included studies.

Study	Demographic characteristics	Balance tasks	Balance equipment/ measures	Main focus/behavioral findings	Quality score
EEG studies					
Bohle et al. (2019) Germany	Total *N* = 58 (YA: 28, OA: 30)	(1) ST semi-tandem stance with EO on foam surface, (2) DT semi-tandem stance with EO on foam surface (spatial one-back task), (3) Cognitive-cognitive-motor task (visual-auditory task and spatial one-back task): semi-tandem stance with EO on foam surface	Force plate: CoP displacement	Dual-tasking 1. OA showed greater CoP displacement across all task conditions than YA. 2. A significant task × group interaction indicated that postural sway increased more with rising task demands in OA than in YA. 3. Cognitive performance was better in the cognitive-motor condition than in the cognitive-cognitive-motor condition in both YA and OA. 4. A significant task × group interaction indicated that cognitive performance was better in the cognitive-cognitive-motor condition in YA than OA, but not in the cognitive-motor condition.	0.77 (Good)
	YA age: 25 ± 3.6 OA age: 71.7 ± 5.4				
	Sex (F/M): 26/32				
Chan et al. (2019) China	*N* = 43	(1) Comfortable stance with EO on firm surface, (2) Narrow stance with EO on firm surface	Force plate: CoP path length, mean sway velocity	Sensory (base of support) 1. Greater path length was observed during narrow stance compared to comfortable stance.	0.82 (Strong)
	Age: 71.3 ± 4.1				
	Sex (F/M): 38/5				
Chang et al. (2016) Taiwan	Total *N* = 31 (LF: 15, HF: 16)	Four continuous and sequential motion phases with and without VR-based scenery: (0) Stationary phase, (1) Slip-forward phase, (2) Pitch-down phase, (3) pitch-up phase	N/A	Sensory (visual) and mechanical challenge N/A	0.64 (Low)
	LF age: 68.4 ± 2.6 HF age: 70.2 ± 2.2				
	Sex (F/M): 19/12				
Chen et al. (2021) Taiwan	*N* = 36	(1) Comfortable stance with EO on foam surface, (2) Comfortable stance with EC on foam surface	Stabilometer inclinometer: 95% confidence ellipse sway area, mean frequency, sample entropy, stabilogram diffusion analysis of CoP	Sensory (visual) challenge 1. EC condition resulted in significant greater postural sway than the EO condition.	0.82 (Strong)
	Age: 66.1 ± 3.1				
	Sex (F/M): 21/15				
Chen et al. (2024) Taiwan	Total *N* = 40 (YA: 20, OA: 20)	Comfortable stance with EO on stabilometer (i.e., movable platform)	Stabilometer inclinometer: RMS of postural sway, sample entropy, mean frequency	Sensory (somatosensory) challenge 1. OA displayed greater postural sway compared to YA.	0.86 (Strong)
	YA age: 24.1 ± 1.9 OA age: 66.2 ± 2.7				
	Sex (F/M): 23/17				
Chen et al. (2025) Taiwan	*N* = 33	Comfortable stance on a stabilometer (i.e., movable platform) with and without intermittent visual interference (i.e., stroboscopic vision)	Stabilometer inclinometer: stabilogram diffusion analysis of CoP	Sensory (visual) challenge 1. Intermittent visual interference resulted in greater postural sway.	0.73 (Good)
	Age: 66.1 ± 2.5				
	Sex (F/M): 18/15				
Chow et al. (2018) China	Total *N* = 79 (YA: 39, OA: 40)	YA: Tandem stance with EO on foam surface OA: Narrow stance with EO on foam surface	3D motion capture: CoM total body sway	Sensory (base of support) challenge N/A	0.77 (Good)
	YA age: 23.5 ± 5.2 OA age: 69.7 ± 3.8				
	Sex (F/M): 47/32				
Chu and Wong (2019) China	*N* = 29	(1) Comfortable stance with EO on foam surface, (2) Narrow stance with EO on foam surface, (3) Tandem stance with EO on foam surface	N/A	Sensory (base of support) challenge 1. Greater task difficulty significantly increased postural sway in OA.	0.73 (Good)
	Age: 75.1 ± 8.4				
	Sex (F/M): 22/7				
Hu et al. (2023) United States	Total *N* = 21 (YA: 10, OA: 11)	(1) Sensory Organization Test (SOT): (1) EO on firm surface (2) EC on firm surface (3) EO with sway-referenced visual surround (4) EO on sway-referenced support surface (5) EC on sway-referenced support surface (6) EC on sway-referenced support surface and surround, (2) Motor control test (MCT), (3) Adaptation test (ADT)	Computerized dynamic posturography: 1) SOT: Equilibrium score, 2) MCT: Latency 3) ADT: Sway energy score	Sensory (visual, somatosensory, visual-somatosensory) and mechanical challenge 1. SOT, MCT, ADT: Significant condition effects indicated the greater postural sway in response to increasing task demands. 2. SOT: There was no age x task interaction effects. 3. MCT, ADT: OA showed greater postural sway than YA.	0.91 (Strong)
	YA age range: 18–30 OA age range: 65–85				
	Sex (F/M): 9/12				
Huang et al. (2017) Taiwan	Total *N* = 24 (YA: 12, OA: 12)	(1) DT comfortable stance with EO on firm surface (force-matching task), (2) DT comfortable stance with EO on stabilometer (unstable) surface (force-matching task)	Ankle movement: RMS and sample entropy	Sensory (somatosensory) challenge and Dual-tasking (motor-motor) 1. A significant age × stance interaction effect indicated greater ankle movement fluctuations during the stabilometer condition in OA compared with YA. 2. A significant age x stance interaction effects indicated worse cognitive performance during stabilometer condition than during firm surface in OA compared to YA.	0.86 (Strong)
	YA age: 25.3 ± 1.3 OA age: 65.8 ± 1.0				
	Sex (F/M): 10/14				
Kahya et al. (2022) United States	*N* = 30	(1) ST comfortable stance with EO on firm surface, (2) DT comfortable stance with EO on firm surface (subtracting 3 s)	Wireless inertial sensors: Mean total sway area, sway velocity, sway path	Dual-tasking N/A	0.91 (Strong)
	Age: 73.1 ± 5				
	Sex (F/M): 13/17				
Li et al. (2022) United States	*N* = 32	(1) Comfortable stance with EO on foam surface, (2) Narrow stance with EO on foam surface, (3) Tandem stance with EO on foam surface, (4) DT tandem stance with EO on foam surface (holding a tray with a plastic cup full of water)	Force plate: Total sway length	Sensory (base of support) challenge and dual-tasking (motor-motor) 1. Increased task demands significantly resulted in greater postural sway. 2. There were no significant differences in postural sway between ST and DT tandem stance conditions.	0.86 (Strong)
	Age: 72.1 ± 4.2				
	Sex (F/M): 25/7				
Lin et al. (2021) Taiwan	Total *N* = 24 (YA: 12, OA: 12)	Sensory organization test (SOT): (1) EO on firm surface (2) EC on firm surface (3) EO with sway-referenced visual surround (4) EO on sway-referenced support surface (5) EC on sway-referenced support surface (6) EO on sway-referenced support surface and surround	Computerized dynamic posturography: Equilibrium score, somatosensory ratio, visual ratio, vestibular ratio	Sensory (visual, somatosensory, visual-somatosensory) challenge 1. OA showed significantly greater postural sway in all conditions compared with YA. 2. The differences in postural sway became pronounced in sway-referenced conditions, particularly for the EC on sway-referenced support surface condition. 3. OA had significantly worse ability in all three sensory ratios.	0.91 (Strong)
	YA age: 22.8 ± 2.6 OA age: 67.7 ± 6.8				
	Sex (F/M): 15/9				
Malcolm et al. (2021) United States	Total *N* = 28 (YA: 14, OA: 14)	Regular stance and tandem stance with and without visual interference	3D motion capture: Mean SD of head velocities	Sensory (visual, somatosensory) challenge 1. A significant stance x group interaction effect indicated greater postural sway in OA as base of support decreased. 2. OA showed significantly greater postural sway during visual interference conditions than YA.	0.86 (Strong)
	YA age: 24.1 ± 3.7 OA age: 76.7 ± 5.2				
	Sex (F/M): 13/15				
Ozdemir et al. (2016) United States	Total *N* = 19 (YA: 10, OA: 9)	2 conditions × ST × non-challenging DT (one-back task) × challenging DT (two-back task): (1) Comfortable stance with EO on firm surface, (2) Comfortable stance with EO on sway-referenced platform	Force plate: estimated CoM sway velocity, Time to boundary (TTB)	Sensory (somatosensory) challenge and dual-tasking 1. OA showed greater DT-related postural sway than YA, when task demands increased. 2. During non-challenging DT conditions, balance performance improved in both YA and OA. 3. During challenging DT conditions, OA showed worse postural sway, whereas YA were less affected by increased cognitive demands.	0.82 (Strong)
	YA age: 26.2 ± 2.8 OA age: 81.4 ± 6.3				
	Sex (F/M): 10/9				
Ozdemir et al. (2018) United States	Total *N* = 19 (YA: 10, OA: 9)	Experiment 1 (1) Comfortable stance with EO on firm surface, (2) Comfortable stance with EC on firm surface, (3) Comfortable stance with EC on sway-referenced platform. Experiment 2: (1) Postural responses to four perturbations: two horizontal translations (backward, forward) and two vertical rotations (toes-up, toes-down)	Force plate: estimated CoM sway angle, Time to boundary (TTB)	Sensory (visual-somatosensory) and mechanical challenge N/A	0.86 (Strong)
	YA age: 26.2 ± 2.8 OA age: 81.4 ± 6.3				
	Sex (F/M): 10/9				
Palmer et al. (2021) United States	*N* = 15	Standing reactive balance test: (1) Postural responses to four perturbations: two horizontal translations (backward, forward) and two vertical rotations (toes-up, toes-down)	Did not measure balance performance during balance tasks. Instead, they used miniBEST total score.	Mechanical challenge 1. Cognitive dual-task interference was strongly associated with reactive balance capacity. 2. MiniBEST scores were also not significantly associated with reactive step threshold.	0.86 (Strong)
	Age: 69 ± 8				
	Sex (F/M): 11/4				
Payne et al. (2021) United States	*N* = 19	Standing reactive balance test: (1) Postural responses to four perturbations: two horizontal translations (backward, forward) and two vertical rotations (toes-up, toes-down)	3D motion capture: CoM displacement, miniBEST total score	Mechanical challenge 1. Poorer cognitive set shifting ability was significantly associated with stiffer balance responses to perturbations.	0.86 (Strong)
	Age: 71 ± 7				
	Sex (F/M): 6/13				
Rubega et al. (2021) Italy	Total *N* = 18 (YA: 9, OA: 9)	2 conditions with and without DT (visual oddball test): (1) Comfortable stance with EO on firm surface (2) Comfortable stance with EO on balance board	Inertial sensor: RoM-AP, ROM-ML, sway path length, the area of the ellipse containing the 95% of the CoM trajectory (CEA)	Sensory (somatosensory) challenge and dual-tasking 1. OA showed greater postural sway during ST and DT conditions compared to YA.	0.77 (Good)
	YA age range: 24–36 OA age range: 64–76				
	Sex (F/M): N/A				
Saadat et al. (2021) Iran	Total *N* = 39 (YA: 19, OA: 20)	Standing reactive balance test: (1) predictable perturbation (eyes open), (2) unpredictable perturbation (eyes closed, earplug)	N/A	Mechanical challenge N/A	0.86 (Strong)
	YA age: 24.3 ± 3.2 OA age: 65.6 ± 4.7				
	Sex (F/M): 16/23				
Tsai et al. (2022) Taiwan	*N* = 36	Comfortable stance on a stabilometer (i.e., unstable surface) with and without intermittent visual interference (i.e., stroboscopic vision)	Stabilometer inclinometer: RMS of postural sway, sample entropy, mean frequency	Sensory (visual, somatosensory) challenge 1. Intermittent visual interference significantly resulted in greater postural sway.	0.91 (Strong)
	Age: 65.8 ± 2.8				
	Sex (F/M): 20/16				
Wang et al. (2025) China	Total *N* = 96 (YA: 49, OA: 47)	(1) VR-based congruent visual condition in comfortable stance with EO on firm surface, (2) VR-based conflict visual condition in comfortable stance with EO on firm surface	Force plate: CoP Postural sway area	Sensory (visual) challenge 1. Visual conflict increased instability in both groups, but the effect was significantly stronger in OA. 2. In the conflict perception task, most OA failed to notice the mismatch, whereas YA were substantially more likely to detect it.	0.86 (Strong)
	YA age: 23 ± 2 OA age: 65 ± 3				
	Sex (F/M): N/A				
Wang et al. (2023) United States	Total *N* = 40 (YA: 20, OA: 20)	Standing reactive balance test: (1) Predictable perturbation, (2) Unpredictable perturbation	N/A	Mechanical challenge N/A	0.91 (Strong)
	YA age: 24.6 ± 6.9 OA age: 66.9 ± 5.8				
	Sex (F/M): 20/20				
Yu et al. (2019) Taiwan	Total *N* = 32 (YA: 16, OA: 16)	Two DT conditions (standing + force-matching task): (1) Postural-focus on tilting platform, (2) Supraposture-focus condition on tilting platform	Balance platform inclinometer: Percentage of determinism	Dual-tasking (motor-motor) 1. Postural sway was greater in OA, and it significantly increased in the postural-focus condition compared to the supraposture-focus condition. 2. Force-matching error increased in the postural-focus condition for both young and older adults, but the increase was larger in OA. 3. OA had greater force-matching error than YA only in the postural-focus condition.	0.86 (Strong)
	YA age: 24.4 ± 5.2 OA age: 69.1 ± 2.7				
	Sex (F/M): N/A				
fNIRS studies					
George et al. (2021) Australia	Total *N* = 49 (YA: 24, OA: 25)	5 conditions with and without DT (subtracting 3 s): (1) Comfortable stance with EO on firm surface, (2) Comfortable stance with EC on firm surface, (3) Narrow stance with EC, (4) Comfortable stance with EC on foam surface, (5) Narrow stance with EC on foam surface	Force plate: mean sway velocity, mean sway frequency, the area of the 95th percentile confidence ellipse of the CoP	Dual-tasking 1. During ST, age-related differences in postural sway became most evident under challenging balance conditions. 2. DT disproportionately impaired postural stability in OA compared with YA when balance demands increased.	0.86 (Strong)
	YA age: 20.8 ± 2.4 OA age: 70.6 ± 7.1				
	Sex (F/M): 30/19				
Hinderaker et al. (2020) United States	Total *N* = 21 (YA: 11, OA: 10)	Comfortable stance on three different optic flow speed conditions (fast:20 m/s, medium: 10 m/s, slow: 5 m/s)	Force plate: CoP, maximum amplitude	Sensory (visual) challenge 1. OA showed increased postural sway compared to YA in the optic flow speed of 10 m/s.	0.82 (Strong)
	YA age: 22 ± 1 OA age: 71 ± 5				
	Sex (F/M): 12/9				
Kan et al. (2025) China	Total *N* = 39 (YA: 19, OA: 20)	3 conditions with and without DT (subtracting 7 s): (1) Comfortable stance with EC on stable plane, (2) Comfortable stance with EO on unstable plane, (3) Comfortable stance with EC on unstable plane	Computerized dynamic posturography: overall stability index (OSI)	Sensory (visual, somatosensory, visual-somatosensory) challenge and dual-tasking 1. Balance performance did not significantly differ between YA and OA. 2. In condition 1, OA showed greater postural sway during DT than during ST. 3. In condition 2, no significant differences between ST and DT were found in OA. 4. In condition 3, significantly lower postural sway was observed during DT than during ST in OA.	0.86 (Strong)
	YA age: 25.1 ± 5.2 OA age: 66.1 ± 4.6				
	Sex (F/M): 23/16				
Lehmann et al. (2022) Germany	Total *N* = 62 (YA: 27, OA: 35)	(1) Comfortable stance with EO on wobble board	Force plate: displacements of CoP	Sensory (somatosensory) challenge 1. OA showed significantly worse balance performance than YA across all balance conditions	1.00 (Strong)
	YA age: 24.8 ± 3.5 OA age: 70.1 ± 4.1				
	Sex (F/M): 27/35				
Lin et al. (2017) United States	Total *N* = 30 (MA: 15, OA: 15)	(1) Comfortable stance with EO in light on firm surface, (2) Comfortable stance with EO in dark on firm surface, (3) Comfortable stance with EO in light on sway-referenced platform, (4) Comfortable stance with EO in dark on sway-referenced platform	Force plate: RMS of CoP data in AP direction	Sensory (visual, somatosensory, visual-somatosensory) challenge 1. No significant group effects were observed across all conditions. 2. Postural sway was greater in the sway-referenced platform conditions than in the firm surface conditions. 3. Changing visual condition did not significantly alter postural sway.	0.77 (Good)
	MA age: 46 ± 11 OA age: 73 ± 5				
	Sex (F/M): 17/13				
Marusic et al. (2019) Slovenia	Total *N* = 20 (YA: 10, OA: 10)	(1) Comfortable stance with EO on firm surface (baseline), (2) ST tandem stance with EO on firm surface, (3) DT tandem stance with EO on firm surface (subtracting 3 s)	Force plate: CoP sway path in AP and ML	Sensory (base of support) challenge and dual-tasking 1. No significant task effects were found in postural sway across conditions and groups. 2. YA showed better cognitive performance during ST and DT than OA.	0.86 (Strong)
	YA age: 22.6 ± 2.8 OA age: 72.3 ± 3.2				
	Sex (F/M): 13/7				
Pan and Tang (2025) United States	Total *N* = 45 (YA: 15, MA: 15 OA: 15)	(1) 10-s baseline of quiet standing with EO on firm surface. (2) four DT conditions (motor task: precision fitting task): opening size (large vs. small) and distance (close vs. far) from the board	Force plate: SD of CoP, mean CoP velocity in AP and ML directions, 95% ellipse of sway area	Dual-tasking (motor-motor) 1. OA showed poorer postural stability than YA and MA. 2. MA showed greater postural sway than YA.	0.82 (Strong)
	YA age: 19.5 ± 0.6 MA age: 48.7 ± 3.1 OA age: 68.0 ± 3.5				
	Sex (F/M): 24/21				
Pan and Zhang (2025) United States	Total *N* = 28 (YA: 14, OA: 14)	(1) ST comfortable stance with EO on firm surface, (2) DT comfortable stance with EO on firm surface (subtracting 7 s)	Force plate: displacements of CoP in AP and ML directions	Dual-tasking 1. There was no significant difference in postural sway in ST conditions between YA and OA, whereas OA showed greater postural sway across DT conditions than YA. 2. OA showed greater DT cost than YA.	0.86 (Strong)
	YA age: 19.8 ± 0.8 OA age: 66.3 ± 3.4				
	Sex (F/M): 14/14				
Rond et al. (2021) Belgium	Total *N* = 34 (YA: 17, OA: 17)	VR-based standing weight-shifting tests with and without DT (subtracting 3 s and 7 s)	3D motion capture: CoM speed, CoM error	Dual-tasking 1. OA performed worse than YA on the weight-shifting task in both ST and DT conditions. 2. For the serial subtraction task, OA showed a higher cognitive DT cost, with significantly worse cognitive performance during DT compared with ST.	0.91 (Strong)
	Median YA age: 22 Median OA age: 77				
	Sex (F/M): 22/12				
Rosso et al. (2017) United States	Total *N* = 16 (YA: 6, OA: 10)	(1) ST comfortable stance with EO on a firm surface, (2) DT comfortable stance with EO on a firm surface; cognitive task: auditory choice reaction time (CRT) task	Computerized dynamic posturography: median absolute deviation (MAD) of the CoM in AP direction	Dual-tasking 1. Reaction time significantly increased in OA than in YA during DT. 2. Postural sway was similar between groups during ST and DT.	0.77 (Good)
	YA age range: 22–30 OA age range: 66–81				
	Sex (F/M): 9/7				
Teo et al. (2018) Australia	Total *N* = 38 (YA: 20, OA: 18)	Sensory organization test (SOT): (1) EO on firm surface (2) EC on firm surface (3) EO with sway-referenced visual surround (4) EO on sway-referenced support surface (5) EC on sway-referenced support surface 6) EO on sway-referenced support surface and surround	Computerized dynamic posturography: CoP sway path in AP and ML	Sensory (visual, somatosensory, visual-somatosensory, vestibular) challenge 1. A significant age × task effect indicate greater postural sway in OA as task demands increased.	0.82 (Strong)
	YA age: 21.5 ± 3.5 OA age: 69.5 ± 3.4				
	Sex (F/M): 16/22				
Xu et al. (2024a) China	Total *N* = 40 (MCI: 21, OA: 19)	(1) ST comfortable stance with EO on firm surface, (2) DT comfortable stance with EO on firm surface (subtracting 3 s)	Force plate: CoP sway path in AP and ML, RMS of AP and ML, 95% confidence ellipse area (95%AREA)	Dual-tasking 1. During DT conditions, the MCI group showed significantly worse postural stability than the control group.	0.95 (Strong)
	MCI age: 71 ± 3 OA age: 70 ± 4				
	Sex (F/M): 21/19				
Xu et al. (2024b) China	Total *N* = 40 (MCI: 21, OA: 19)	(1) Comfortable stance with EO on firm surface, (2) Comfortable stance with EO on foam surface, (3) Comfortable stance with EC on firm surface, (4) Comfortable stance with EC on foam surface	Force plate: CoP sway path in AP and ML, RMS of AP and ML, 95% confidence ellipse area (95%AREA)	Sensory (visual, somatosensory, visual-somatosensory) challenge 1. MCI group showed greater postural sway than the control group across all conditions. 2. Greater postural sway was found on foam surface than on firm surface in MCI group.	0.86 (Strong)
	MCI age: 71 ± 3 OA age: 70 ± 4				
	Sex (F/M): 21/19				

LF, low fall risk group; HF, high fall risk group; YA, young adults; MA, middle-aged adults; OA, older adults; MCI, mild cognitive impairment; EO, eyes open; EC, eyes closed; ST, single-task; DT, dual-task; CoP, center of pressure; CoM, center of mass; RMS, root-mean-square; RoM, range of motion; AP, anteroposterior; ML, mediolateral.

### Participants characteristics

A total of 1,302 individuals, including 788 OA (60.5%), 30 MA (2.3%), and 484 YA (37.2%), were included in this review. The median total sample size of the included studies was 32 (range: 15–95), and the median OA sample size was 20 (range: 9–46). Most participants were female (*n* = 622, 56.1%), with the mean age of YA ranging from 19 to 26, MA ranging from 46 to 49, and OA ranging from 65 to 81.

Out of 37 studies, 25 studies recruited healthy YA (*n* = 22) (Ozdemir et al., 2016; Huang et al., 2017; Rosso et al., 2017; Chow et al., 2018; Ozdemir et al., 2018; Teo et al., 2018; Bohle et al., 2019; Marusic et al., 2019; Yu et al., 2019; Hinderaker et al., 2020; George et al., 2021; Lin et al., 2021; Malcolm et al., 2021; Rond et al., 2021; Rubega et al., 2021; Saadat et al., 2021; Lehmann et al., 2022; Hu et al., 2023; Wang et al., 2023; Chen et al., 2024; Kan et al., 2025; Pan and Zhang, 2025; Wang et al., 2025) or MA (*n* = 2) (Lin et al., 2017; Pan and Tang, 2025) as a control group. Of the 12 studies (Chang et al., 2016; Chan et al., 2019; Chu and Wong, 2019; Chen et al., 2021; Palmer et al., 2021; Payne et al., 2021; Kahya et al., 2022; Li et al., 2022; Tsai et al., 2022; Xu et al., 2024a; Xu et al., 2024b; Chen et al., 2025) that recruited only healthy OA, two studies (Xu et al., 2024a; Xu et al., 2024b) additionally included OA with mild cognitive impairment (MCI). Interestingly, Chang et al. (2016) assigned OA participants into high- or low-fall risk groups. Detailed methodological characteristics of included studies are provided in Table 1.

### Characteristics of included studies

All studies employed a cross-sectional design. Most studies were conducted in the United States (*n* = 14, 38.9%), followed by Taiwan (*n* = 8, 22.2%) and China (*n* = 7, 19.4%). Notably, all included studies were published within the past 10 years, spanning from 2016 to 2025.

Included studies employed various perturbation methods during standing balance tasks. Most studies manipulated visual inputs, either by removing visual information (i.e., instructing participants to close their eyes, *n* = 6) (Ozdemir et al., 2018; Teo et al., 2018; Chen et al., 2021; George et al., 2021; Lin et al., 2021; Hu et al., 2023) or by providing visual interference such as optic flow imagery (*n* = 6) (Chang et al., 2016; Hinderaker et al., 2020; Malcolm et al., 2021; Tsai et al., 2022; Chen et al., 2025; Wang et al., 2025). Studies increased postural demands by using a foam surface to disrupt proprioception (*n* = 5) (Chow et al., 2018; Bohle et al., 2019; Chu and Wong, 2019; Chen et al., 2021; George et al., 2021; Li et al., 2022), decreasing base of support by altering preferred stance (e.g., semi-tandem or tandem position; *n* = 7) (Chow et al., 2018; Chan et al., 2019; Chu and Wong, 2019; Marusic et al., 2019; George et al., 2021; Lin et al., 2021; Malcolm et al., 2021), and including balance board/movable platforms (*n* = 8) (Huang et al., 2017; Yu et al., 2019; Rubega et al., 2021; Lehmann et al., 2022; Tsai et al., 2022; Chen et al., 2024; Chen et al., 2025; Pan and Tang, 2025), or sway-referenced platforms (*n* = 6) (Ozdemir et al., 2016; Lin et al., 2017; Ozdemir et al., 2018; Teo et al., 2018; Lin et al., 2021; Hu et al., 2023). Thirteen studies (Chang et al., 2016; Lin et al., 2017; Ozdemir et al., 2018; Teo et al., 2018; Chen et al., 2021; George et al., 2021; Lin et al., 2021; Malcolm et al., 2021; Tsai et al., 2022; Hu et al., 2023; Xu et al., 2024b; Chen et al., 2025; Kan et al., 2025) perturbed two or more sensory inputs simultaneously (e.g., standing with eyes closed on a foam surface). Fourteen studies used dual-tasking during standing balance tasks, either cognitive-motor (*n* = 10) (Ozdemir et al., 2016; Rosso et al., 2017; Bohle et al., 2019; Marusic et al., 2019; George et al., 2021; Rond et al., 2021; Rubega et al., 2021; Kahya et al., 2022; Xu et al., 2024a; Kan et al., 2025; Pan and Zhang, 2025) or motor-motor (*n* = 4) (Huang et al., 2017; Yu et al., 2019; Li et al., 2022; Pan and Tang, 2025), combined with visual and/or somatosensory manipulation. Six studies mechanically perturbed participants’ balance (Ozdemir et al., 2018; Palmer et al., 2021; Payne et al., 2021; Saadat et al., 2021; Hu et al., 2023; Wang et al., 2023). The median standing time for each trial, except for studies using mechanical challenges, was 40 s (range: 20–121).

Twenty-four studies (64.9%) employed EEG methodologies. Twelve studies (Chang et al., 2016; Ozdemir et al., 2016; Ozdemir et al., 2018; Bohle et al., 2019; Lin et al., 2021; Malcolm et al., 2021; Palmer et al., 2021; Rubega et al., 2021; Saadat et al., 2021; Kahya et al., 2022; Tsai et al., 2022; Hu et al., 2023) used the power spectrum of frequency bands as an outcome, with the beta (12–30 Hz, *n* = 12) and alpha (8–12 Hz, *n* = 10) bands assessed most frequently, followed by theta (4–8 Hz, *n* = 8), gamma (30–50 Hz, *n* = 5), and delta (0.5–4 Hz, *n* = 4) bands. Two studies (Chen et al., 2024; Chen et al., 2025) assessed PAC, an index of cortico-posture coupling. Several studies assessed functional connectivity in various metrics, including the Phase-Lag Index (PLI) (Chen et al., 2021; Tsai et al., 2022) and ERP using the synchronization likelihood (SL) (Huang et al., 2017). Six studies also assessed functional connectivity using coherence analyses, with four studies measuring T3–Fz (i.e., verbal-analytical with motor planning) and/or T4–Fz (i.e., visuospatial with motor planning) coherence, defined according to the international 10–20 system (Jasper, 1958), at fast alpha frequency (10–12 Hz). Saadat et al. (2021) measured F3–C3, F3–P3, C3–P3, F4–C4, F4-P4, and C4–P4 coherence at alpha and beta bands. Palmer et al. (2021), who employed mechanical perturbation, assessed perturbation-evoked coherence at the beta band over Cz–CPz (i.e., motor cortex–primary somatosensory cortex) and Cz–AFz (i.e., motor cortex–prefrontal cortex). Four studies measured ERPs after mechanical perturbations using N1 and P1 latencies (Ozdemir et al., 2018), N1 and P2 amplitudes (Huang et al., 2017), only N1 amplitude (Payne et al., 2021), and root-mean-square (RMS) amplitude (Yu et al., 2019). Here, N and P denote negative and positive ERP components, respectively. Of note, Yu et al. (2019) assessed ERPs of P1, N1, and P2 amplitudes during motor-motor standing (i.e., standing while force-matching task) balance tasks. In terms of RoIs in EEG studies, frontal areas (*n* = 21) were most frequently assessed, followed by central (*n* = 18), parietal (*n* = 17), temporal (*n* = 12), and occipital (*n* = 11) areas. Table 2 provides detailed information regarding cortical activity measures, RoIs, and main cortical activity findings in each study.

**TABLE 2:** Summary of brain measures, regions of interest, and cortical activation findings.

Study	Cortical activity measures	Regions of interest	Cortical activity findings	Association between cortical activity and postural sway
EEG studies				
Bohle et al. (2019) Germany	Absolute PSD: delta (0.5–4 Hz), theta (4–7.5 Hz), alpha (8–12 Hz), and beta (13–30 Hz) bands	Anterior midline (Fz, FCz), central midline (Cz), posterior midline (Pz, POz), anterior (F7/8, F5/6, F3/4, FT7/8, FC5/6, FC3/4), central (C3/4, C5/6, T7/8, CP3/4, CP5/6, TP7/8), posterior (P7/8, P5/6, P3/4, PO7/8, PO5/6, PO3/4)	1. Delta and theta power increased with task difficulty in both YA and OA across brain areas, with frontal areas being most pronounced. 2. YA compared to OA showed higher delta activities at the mid-anterior (Fz) electrode during balance tasks. 3. YA compared to OA showed higher theta activities at midline electrodes (Fz, Cz, Pz) during balance tasks. 4. Greater decreases were shown in alpha activity with increased task difficulty in YA and OA, with greater decrease shown in YA. 5. There were no group and task effects in beta activity.	N/A
Chan et al. (2019) China	T3-Fz and T4-Fz coherence at fast alpha (10–12 Hz) band	Fz, T3, T4	N/A	1. Higher MSRS-C (i.e., higher tendency for conscious movement) scores were significantly associated with smaller changes in T3-Fz coherence when participants shifted from wide to narrow base (*B* = -0.49, *p* < 0.001). 2. No significant correlations were observed between T4-Fz coherence and scores on the MSRS-C or FES-I.
Chang et al. (2016) Taiwan	Relative PSD: theta (4–7 Hz), alpha (8–12 Hz), beta (12–30 Hz), and gamma (30–40 Hz) bands	Fz, Cz, Pz, Oz	1. Visual interference resulted in significantly higher PSD at Fz, Cz, Pz, and Oz in both LF and HF groups. 2. Theta, beta, and gamma power significantly increased with visual interference at Fz and Oz across balance tasks, while alpha power did not change. 3. The LF group showed higher theta and alpha power at Cz and Oz compared to the HF group during the SF phase. 4. The HF group showed higher PSD in all wave bands at Pz than the LF group during recovery phase, particularly under visual perturbation.	1. There was a significant and strong correlation between posture-related cortical regions (Fz–Cz, Fz–Pz, Cz–Oz, Cz–Pz, Pz–Oz) in the LF group, but not in the HF group, during the platform recovery phase under visual interference.
Chen et al. (2021) Taiwan	Functional connectivity: phase-lag index (PLI) and minimum spanning tree (MST) metrics at delta (1–3 Hz), theta (4–7 Hz), alpha (8–12 Hz), and beta (13–35 Hz) bands	Fp1/2, Fz, F3/4, F7/8, FT7/8, FCz, FC3/4, Cz, C3/4, CPz, CP3/4, Pz, P3/4, T3/4, T5/6, TP7/8, Oz, and O1/2	1. EC weakened fronto-central connectivity but strengthened fronto-parietal-occipital connectivity, especially in theta, alpha, and beta bands.	1. In the EO condition, postural sway was significantly correlated with cortical network in the delta, theta, and beta bands. 2. In the EC condition, no such correlations were observed.
Chen et al. (2024) Taiwan	Phase-amplitude coupling (PAC) between postural sway and the amplitude of sub-band EEG: theta (4–7 Hz), alpha (8–12 Hz), and beta (13–35 Hz) bands	Fp1/2, Fz, F3/4, F7/8, FT7/8, FCz, FC3/4, Cz, C3/4, CPz, CP3/4, Pz, P3/4, T3/4, T5/6, TP7/8, Oz, and O1/2	1. YA showed a greater synchronization between theta activity and postural sway than OA, particularly over FCz and bilateral temporal-parietal-occipital regions. 2. YA showed greater synchronization between alpha activity and postural sway than OA, particularly in temporal, parietal, and occipital regions (T5, O1, CPz, TP8, P4, T6). 3. OA showed greater synchronization between beta activity and postural sway over the left primary motor cortex (C3) than YA.	N/A
Chen et al. (2025) Taiwan	Phase-amplitude coupling (PAC) between postural sway and the amplitude of sub-band EEG: theta (4–7 Hz), alpha (8–12 Hz), and beta (13–35 Hz) bands	Fp1/2, Fz, F3/4, F7/8, FT7/8, FCz, FC3/4, Cz, C3/4, CPz, CP3/4, Pz, P3/4, T3/4, T5/6, TP7/8, Oz, and O1/2	1. Intermittent visual interference significantly enhanced theta-band PAC (4–7 Hz) compared to the EO condition, mainly in fronto-motor (Fp1/2, F7/8, F3/4, Fz, FT7/8, FC3/4, FCz, T3/4, C3/4, Cz) and parietal regions (P3, CP3). 2. Intermittent visual interference increased alpha-band PAC (8–12 Hz) compared to the EO condition over bilateral fronto-motor and parietal regions. 3. Intermittent visual interference increased beta-band PAC compared to EO condition, particularly over frontal (Fp1/2, F3/4, Fz, F7/8) and sensorimotor regions (FC3/4, FCz, T3/4, C3/4, CP3).	1. Significant and negative correlations were found between normalized changes in short-term open-loop control and normalized changes in beta-band PAC in the frontal (*r* = -0.41, *p* = 0.02) and sensorimotor (*r* = -0.44, *p* = 0.01) regions.
Chow et al. (2018) China	T3-Fz coherence at fast alpha (10–12 Hz) band	Motor planning (Fz), verbal-analytical (T3)	1. Significant changes in T3–Fz coherence and body sway were observed from baseline to internal focus in YA. 2. No significant changes in T3–Fz coherence or body sway were observed from baseline to internal focus in OA.	1. No significant correlations were observed between the percentage change in T3–Fz coherence and total body sway from Baseline to Internal focus in YA (*r* = 0.09, *p* = 0.30) and OA (*r* = 0.13, *p* = 0.22). 2. MSRS-C scores were not correlated with Baseline T3–Fz coherence in YA (*r* = 0.11, *p* = 0.26) and OA (*r* = -0.05, *p* = 0.38).
Chu and Wong (2019) China	T3-Fz and T4-Fz coherence at fast alpha (10–12 Hz) band	Motor planning (Fz), verbal-analytical (T3), visuospatial (T4) regions	1. T3–Fz EEG coherence at tandem stance on foam surface was significantly higher than at narrow stance on foam surface (*p* = 0.03) and comfortable stance on foam surface (*p* = 0.02). 2. There were no significant differences in T4–Fz coherence across the three standing positions.	N/A
Hu et al. (2023) United States	Relative PSD: beta (13–30 Hz) band	Fz, Cz, Pz	1. Under sensory manipulation (SOT), OA demonstrated significantly higher beta power across all postural control-related cortical areas compared to YA. 2. Compared to eyes-open, both YA and OA showed significantly lower relative beta power in eyes-closed condition. 3. Compared to EO on firm condition, both YA and OA showed lower relative beta power in EC on sway platform condition. 4. YA showed increased relative beta power as tasks became more challenging, while OA decreased relative beta power. 5. Under rapid mechanical perturbation (MCT), OA demonstrated significantly higher relative beta power at Cz than YA.	1. SOT: Age subgroup analysis revealed no correlation in OA, but a significant moderate negative correlation between beta power and lower equilibrium score (i.e., greater sway) in YA at Pz (ρ = -0.436, *p* < 0.001). 2. MCT: In OA, relative beta power was positively correlated with average latency (i.e., worse balance) at Fz (ρ = 0.299, *p* < 0.05) and Cz (ρ = 0.315, *p* < 0.05); no correlations were observed in YA.
Huang et al. (2017) Taiwan	ERP: amplitude of N1 (80–150 ms after signal onset) and P2 (150–240 ms after signal onset), functional connectivity of ERP using synchronization likelihood (SL) method over all brain regions and specific areas of fronto-sensorimotor and parietal-occipital regions.	Frontal (GF: Fp1, Fp2, F3, Fz, F4, F7, and F8), sensorimotor (SM: C3, Cz, C4, CP3, CPz, and CP4), and parietal-occipital (PO: P3, Pz, P4, O1, Oz, and O2)	1. YA showed greater N1 amplitude than OA over frontal and sensorimotor regions, but not in parietal-occipital regions. 2. P2 amplitudes were higher on the stabilometer (dynamic) surface than on the firm surface in both YA and OA. 3. There were no significant differences in P2 amplitude between YA and OA in any brain region. 4. Overall functional connectivity was greater in the stabilometer condition than in the firm surface condition for both YA and OA. 5. OA enhanced functional connectivity in the fronto-sensorimotor cortex and between the frontal and prefrontal cortex as balance demands increased. 6. OA increased parietal-occipital connectivity as postural demands increased, whereas YA showed decreased connectivity in these regions.	N/A
Kahya et al. (2022) United States	Absolute PSD: theta (4–7 Hz), alpha (8–16 Hz), beta (18–32 Hz), and theta/beta power ratio	Anterior (F7/8, Fp1/2, F3/4, FC1/2, FC5/6, AF3/4), central (C3/4, CP1/2, CP5/6, T7/8), posterior (P7/8, P3/4, PO1/2, PO3/4)	1. Compared to quiet standing, DT induced a significant reduction in alpha power in the central-left, central-right, and posterior-left regions. 2. OA exhibited a significant increase in theta power and theta/beta ratio across all cortical regions during DT compared to ST. 3. No significant changes were observed in beta power across cortical regions between ST and DT.	1. OA who exhibited a greater increase in the theta/beta power ratio in the anterior-left (*r* = 0.52, *p* < 0.01), central-right (*r* = 0.53, *p* < 0.01), and posterior-left (*r* = 0.45, *p* < 0.01) regions showed a greater dual-task cost of postural sway. 2. OA who exhibited greater alpha power in the anterior-right (*r* = 0.52, *p* < 0.01) and central-right (*r* = 0.48, *p* < 0.01) regions had greater postural sway during DT.
Li et al. (2022) United States	T3-Fz and T4-Fz coherences at fast alpha (10–12 Hz) frequency	T3, T4, Fz, Fp1	1. No significant differences in T3-Fz and T4-Fz coherence were observed across balance tasks with different base of support positions. 2. No significant differences were found in T3-Fz and T4-Fz coherence between ST tandem stance and DT tandem stance.	
Lin et al. (2021) Taiwan	Event-related spectral perturbation: alpha (8–12 Hz), beta (13–21 Hz), and fast beta (22–30 Hz) bands	Sensory association areas: Bilateral temporal, parietal, and occipital areas	1. Compared to the EO on firm surface condition, both YA and OA showed increased alpha and beta power in the EC on firm and EC on foam conditions across all RoI clusters. 2. YA showed significantly greater increase in alpha and beta power than OA in the EC on firm and EC on foam conditions compared to EO on firm surface condition across all RoI clusters. 3. In somatosensory manipulation conditions, alpha and fast beta power significantly decreased in YA, while minimal change was found in OA, across temporal-occipital, parietal-temporal-occipital, and occipital areas. 4. Across most conditions, OA appeared to have higher fast beta activity than YA in all cortical areas. However, there were no changes in fast beta activity with visual and somatosensory manipulations in OA. 5. OA showed blunted EEG responses to sensory manipulations, particularly in alpha and beta bands, compared to YA.	N/A
Malcolm et al. (2021) United States	Absolute PSD: theta (3–7 Hz), alpha (8–12 Hz), beta (13–30 Hz), and gamma (31–45 Hz) bands	sensorimotor frontal and parietal areas: SMA, precentral gyrus cluster, postcentral gyrus cluster, precuneus cluster	1. Optic flow (visual interference) significantly reduced alpha and beta power in SMA and left precuneus in YA and OA. 2. Only OA showed a reduction in theta power associated with optic flow compared to static visual display in the left precuneus cluster. 3. Tandem stance significantly increased theta power in the SMA compared to normal stance in both YA and OA, while increased power during tandem stance was more pronounced for YA compared to OA. 4. OA showed significant alpha/beta power reductions during tandem stance compared to normal stance in SMA, left/right precentral cluster, and left precuneus cluster, whereas YA showed little change. 5. Only YA but not OA showed an increase in gamma power during tandem stance compared to regular stance.	1. Only significant correlations were observed between mediolateral sway in the YA and beta power in the right frontal cluster (*r* = 0.67, *p* = 0.048) and the left parietal cluster (*r* = 0.66, *p* = 0.036). 2. No significant correlations were found between postural sway and cortical activity in the OA.
Ozdemir et al. (2016) United States	Relative PSD: delta (1–4 Hz), theta (4–7 Hz), alpha (8–12 Hz), beta (14–24 Hz), and gamma (30–50 Hz) bands	Frontal (F3, F1, Fz, F2, F4), central-frontal (FC5, FC3, FC1, FC2, FC4, FC6), central (C3, C1, Cz, C2, C4), central-parietal (CP3, CP1, CPz, CP2, CP4) and parietal (P3, P1, Pz, P2, P4) cortices	1. Delta power increased during DT involving challenging postural control in the frontal, central-frontal, central, and central-parietal regions. 2. Theta power increased during dual tasks with higher cognitive load, especially over frontal and central-frontal regions. 3. YA had higher delta and theta activity than OA over the frontal and central-frontal areas across most balance conditions. 4. Alpha activity increased with greater postural challenge, particularly in parietal and occipital regions. 5. There were no group and condition effects in beta activity. 6. OA but not YA exhibited increased gamma power during DT with challenging stance over frontal and central-parietal regions.	N/A
Ozdemir et al. (2018) United States	Exp 1: Relative PSD: delta (0.1–4 Hz), theta (4–7 Hz), alpha (8–12 Hz), beta (14–24 Hz), and gamma (30–50 Hz) bands Exp 2: Perturbation evoked cortical potentials (PEP): P1 and N1 latencies, and P1-N1 amplitude differences	Frontal (F3, F1, Fz, F2, F4), central-frontal (Fc5, Fc3, Fc1, Fc2, Fc4, Fc6), central (C3, C1, Cz, C2, C4), central-parietal (Cp3, Cp1, Cpz, Cp2, Cp4) and parietal (P3, P1, Pz, P2, P4) cortices.	1. YA had significantly higher delta power during SEC at central cortices and during UEC at central and central-parietal cortices compared to OA. 2. OA showed significantly higher gamma power during UEC conditions in central-parietal, central, and frontal cortices compared to YA. 3. Significantly increased gamma activity was found from SEO to UEC postural conditions in central-frontal and central-parietal cortices. 4. No significant EEG power modulations were observed in alpha, theta and beta bands across postural conditions. 5. OA had significantly longer PEP latencies, including P1 and N1, primarily over central and central-parietal cortices, compared to YA. 6. YA showed significantly greater N1 amplitude than OA over the central cortices. 7. No significant differences were found in P1 response amplitudes between YA and OA.	1. During challenging balance tasks, high delta power was significantly correlated with higher TTB (i.e., lower postural sway; *r* = 0.25–0.33, *p* < 0.05) in OA. 2. EEG power showed very weak correlations with postural sway metrics across all frequency bands and groups, indicating little to no relationship between cortical activity and postural control under stable balance conditions.
Palmer et al. (2021) United States	Beta oscillatory power in response to balance perturbations Perturbation-evoked beta coherence: motor cortex (Cz) with primary somatosensory (CPz), motor cortex (Cz) with prefrontal cortical (Afz) regions.	Motor cortex (Cz), primary somatosensory (CPz), prefrontal cortical (Afz) regions	1. Balance perturbations exhibited significant increases in motor cortical beta power in OA. 2. Balance perturbations did not significantly alter somatosensory (Cpz)–motor (Cz) or prefrontal (Afz)–motor (Cz) beta coherence in OA.	1. Greater late-phase (300–500 ms) perturbation-evoked motor cortical beta power was negatively correlated with miniBEST score (*r* = –0.56, *p* = 0.04). 2. There was no relationship between miniBEST score and either somatosensory–motor or prefrontal–motor beta coherence.
Payne et al. (2021) United States	Cortical N1 response amplitudes between 100 and 200 ms after perturbation onset.	Cz	N/A	1. The miniBEST score was not significantly associated with the peak amplitude of cortical N1 responses. 2. Lower cognitive set-shifting ability was associated with larger perturbation-evoked cortical N1 responses (*p* < 0.05).
Rubega et al. (2021) Italy	Relative PSD: delta (1–4 Hz), theta (4–8 Hz), alpha (8–12 Hz), beta (14–24 Hz), low gamma (30–50 Hz), and high gamma (50–70 Hz) bands	45 brain cortices, including frontal, parietal, temporal, and occipital regions	1. OA showed greater delta activity over frontal regions during standing on firm surface, whereas YA exhibited higher alpha, beta, and gamma activity over frontal, sensorimotor, and occipital areas. 2. During standing on a balance board, OA had higher theta power than YA. 3. In OA, DT standing on a balance board elicited stronger sensorimotor alpha/beta and frontal high-gamma band activities compared with DT standing on firm surface. 4. OA compared to YA showed higher frontal and occipital theta, as well as frontal gamma power, during DT.	N/A
Saadat et al. (2021) Iran	Absolute PSD: alpha (8–12 Hz) and beta (12.5–25 Hz) bands F3–C3, F3–P3, C3–P3, F4–C4, F4-P4, and C4– P4 coherence at alpha and beta bands	C3, C4, F3, F4, P3, P4.	1. In the predictable condition, OA exhibited higher beta power and higher alpha and beta coherence during compensatory postural adjustment period than YA. 2. In unpredictable condition, YA showed higher alpha power in P4 than OA. 3. OA displayed significantly lower alpha power during anticipatory postural adjustment period and higher beta power during the compensatory phase than YA. 4. In unpredictable condition, OA showed higher alpha coherence in the F3-P3 and F4-P4 regions, and higher beta coherence in the F4-P4 region compared to YA.	N/A
Tsai et al. (2022) Taiwan	Relative PSD: theta (4–7 Hz), alpha (8–12 Hz), and beta (13–30 Hz) bands functional connectivity: phase-lag index (PLI) and minimum spanning tree (MST) metrics at theta, alpha, and beta bands.	N/A	1. Intermittent visual interference on a stabilometer reduced theta power in frontal (Fz) and left sensorimotor regions (FC3, C3, CP3) but increased alpha power in fronto-centro-parietal areas (FC2, Cz, CPz, Pz) and beta power over fronto-centro-parietal and occipital areas (O1, O2, Oz). 2. Intermittent visual interference strengthened functional connectivity during standing balance in alpha and beta bands.	1. Larger visual interference-related changes in mid-frontal theta power were associated with larger postural sway (*r* = 0.36, *p* = 0.04). 2. Participants with greater normalized changes in postural mean frequency also tended to have stronger Fp2 alpha-band connectivity sway (*r* = 0.34, *p* = 0.049).
Wang et al. (2025) China	Generalized partial directed coherence on mu rhythm (8–13 Hz)	Bilateral somatosensory (S1), motor (MC), posterior parietal (PPC), and visual cortices (VC)	1. YA displayed stronger connectivity within visual integration networks under sensory-congruent conditions. 2. Under sensory-conflict conditions, YA exhibited enhanced connectivity within somatosensory and motor integration networks, indicating a shift toward reliance on body-centered information when visual cues were unreliable. 3. Under sensory-conflict conditions, OA showed a more uniform pattern of connectivity across both conditions.	1. In OA, under the sensory-congruent condition, motor cortex efficiency is correlated with greater postural sway (*r* = 0.55, *p* < 0.001), whereas visual cortex efficiency was not significantly related to postural sway (*r* = -0.05). 2. In OA, under the sensory-conflict condition, visual cortex efficiency is correlated with reduced postural sway (*r* = -0.33, *p* = 0.034), while motor cortex efficiency was not significantly associated with sway (*r* = 0.18).
Wang et al. (2023) United States	RMS amplitude during the 300 ms after perturbation	Cz, FCz	1. Unpredictable perturbation produced significantly greater RMS amplitude at Cz and Fz than predictable disturbances. 2. OA showed greater RMS amplitude than YA at Cz.	
Yu et al. (2019) Taiwan	ERP: P1 (70–110 ms after signal onset), N1 (100–170 ms after signal onset), and P2 (170–270 ms after signal onset) peak amplitude	frontal (Fz, F3, F4, FCz, FC3, and FC4), sensorimotor-parietal (Cz, C3, C4, CPz, CP3, CP4, Pz, P3, and P4), frontotemporal (F7/8, FT7/8, and T3/4) areas	1. Only OA showed visible P1 wave during postural-focus (PF) and suprapostural-focus (SF) tasks, especially over sensorimotor-parietal and fronto-temporal regions. 2. Both YA and OA showed increased N1 peaks in the sensorimotor-parietal regions during PF compared to SF. 3. YA showed larger P2 peaks than OA across both balance conditions. 4. YA increased P2 peaks from SF to PF over the sensorimotor-parietal regions, whereas OA showed decreased P2 peaks. 5. OA showed greater P2 peaks in the right fronto-temporal region from SF to PF.	N/A
fNIRS studies				
George et al. (2021) Australia	Oxygenated hemoglobin (HbO_2_)	PFC	1. HbO_2_ levels were higher in OA compared to YA during ST. 2. HbO_2_ levels significantly increased as the balance conditions became more challenging. 3. In DT conditions, particularly under difficult DT conditions, HbO_2_ levels significantly decreased in OA. 4. In DT conditions, HbO_2_ levels were significantly elevated in YA.	N/A
Hinderaker et al. (2020) United States	Oxygenated (HbO_2_) and deoxygenated hemoglobin (HHb)	Dorsolateral PFC, temporoparietal area (VEST)	1. OA showed an increased tendency of HbO_2_ levels in the DLPFC and VEST during slow and medium optic flow speeds compared to YA. 2. HbO_2_ activity in the DLPFC and VEST significantly decreased during fast optic flow speed in OA compared to YA.	N/A
Kan et al. (2025) China	Oxygenated hemoglobin (HbO_2_)	Bilateral SMC, PMC, PFC	1. YA showed increased HbO_2_ levels only in the LPFC and RPFC during DT in condition 1 (comfortable stance with EC on stable plane task). 2. YA showed increased HbO_2_ levels only in the LSMC during DT in condition 2 (comfortable stance with EO on unstable plane). 3. OA showed increased HbO_2_ levels across all ROIs during DT in conditions 1 and 2. 4. OA showed heightened HbO_2_ levels in the RSMC during DT in condition 3 (comfortable stance with EC on unstable plane).	N/A
Lehmann et al. (2022) Germany	Oxygenated (HbO_2_) and deoxygenated hemoglobin (HHb)	SFG, SFGmedial, MFG, SMA, PreCG, PoCG, MTG	1. OA showed higher HbO_2_ responses in the dorsolateral and medial superior frontal gyrus (SFG) compared to YA during standing on a wobble board. 2. OA showed lower HbO_2_ responses in the postcentral gyrus (PoCG) compared to YA during standing on a wobble board.	1. Lower activation in the prefrontal regions (dorsolateral and medial SFG, and MFG) is linked to better balance performance across age groups. 2. Activity in the dorsolateral SFG mediates the relationship between age and balance performance.
Lin et al. (2017) United States	Oxygenated (HbO_2_) and deoxygenated hemoglobin (HHb)	Frontal-lateral, temporal-parietal, occipital regions	1. Both MA and OA showed increased HbO_2_ levels across nearly all ROIs when sensory input became more limited. 2. OA exhibited greater HbO_2_ levels in the occipital region compared to MA. 3. The next most frequently activated regions in OA were the frontal–lateral (attention) and temporal–parietal (vestibular) regions.	N/A
Marusic et al. (2019) Slovenia	Oxygenated (HbO_2_) and deoxygenated hemoglobin (HHb)	PFC	1. A significant increase in PFC HbO_2_ levels were observed during ST and DT tandem stance compared to quiet standing in both YA and OA. 2. There were no significant differences in PFC HbO_2_ levels between ST and DT tandem stance in both YA and OA. 3. No age-related differences in PFC HbO_2_ levels were found between YA and OA.	N/A
Pan and Tang (2025) United States	Oxygenated hemoglobin (HbO_2_)	Bilateral dorsolateral, ventrolateral, dorsomedial PFC	1. OA showed greater HbO_2_ levels than YA in all PFC regions across DT balance tasks. 2. OA exhibited greater HbO_2_ concentration than MA over left dorsolateral and ventrolateral PFC across DT balance tasks.	N/A
Pan and Zhang (2025) United States	Oxygenated hemoglobin (HbO_2_)	Bilateral dorsolateral, ventrolateral, dorsomedial PFC	1. DT caused significantly greater HbO_2_ activation over dorsolateral PFC in YA, whereas dorsolateral PFC activity did not change between ST and DT in OA. 2. In both YA and OA, DT elicited greater HbO_2_ activation in medial and ventrolateral PFC regions.	1. In ST condition, greater PFC HbO_2_ levels were correlated with larger postural sway in OA. 2. In DT condition, no significant correlations were found in OA. 3. In both ST and DT, YA showed significantly positive correlations between PFC HbO_2_ levels and postural sway.
Rond et al. (2021) Belgium	Oxygenated (HbO_2_) and deoxygenated hemoglobin (HHb)	Bilateral SMA, PMC, SSC, PFC, FEF	1. OA displayed higher HbO_2_ levels in the right SMA and right SSC compared to YA across both ST and DT. 2. OA showed higher HbO_2_ levels in the left PFC than YA during the weight-shifting standing balance task. 3. In DT compared to ST, HbO_2_ levels significantly decreased in OA in the left PFC and left FEF.	1. Higher PFC HbO_2_ values were positively correlated with the number of wasps hit during the game (*r* = 0.47, *p* = 0.03), indicating better balance performance. 2. DT performance was not correlated with HbO_2_ levels.
Rosso et al. (2017)	Oxygenated (HbO_2_) and deoxygenated hemoglobin (HHb)	Left PFC, superior temporal gyrus (STG), supra-marginal gyrus (SMG)	1. OA exhibited significantly more widespread HbO_2_ levels, including PFC, SMG, and STG, during ST balance task compared to YA. 2. During DT, both OA and YA showed significantly reduced HbO_2_ levels relative to ST.	N/A
Teo et al. (2018) Australia	Oxygenated (HbO_2_) and deoxygenated hemoglobin (HHb)	Dorsolateral PFC	1. OA showed a significantly greater increase in HbO_2_ levels compared with YA across all balance conditions. 2. OA exhibited greater changes in HbO_2_ during Conditions 2–6 compared to condition 1 (EO on firm surface), whereas YA showed increases only during conditions 3–6.	1. For sensory Conditions 1 and 2, there was no association between changes in HbO_2_ levels and equilibrium scores. 2. For sensory Conditions 3–6, a greater increase in HbO_2_ levels was associated with higher equilibrium scores (i.e., better balance performance) in both YA and OA.
Xu et al. (2024a) China	Oxygenated hemoglobin (HbO_2_)	Bilateral frontopolar, dorsolateral, ventrolateral, and orbitofrontal PFC	1. OA with MCI showed significantly greater HbO_2_ levels than healthy OA during both ST and DT. 2. There were no significant task or task × group interaction effects.	1. During ST, lower PFC activation was correlated with greater postural sway in OA with MCI (*r* = -0.49 to -0.47, *p* < 0.05). 2. During DT, greater PFC activation was correlated with greater postural sway in OA with MCI (*r* = 0.45–0.56, *p* < 0.05). 3. During ST, positive correlations were found between PFC HbO_2_ levels and postural sway during ST in OA without MCI. 4. During DT, no correlations were found between PFC HbO_2_ levels and balance performance in OA without MCI.
Xu et al. (2024b) China	Oxygenated hemoglobin (HbO_2_)	Bilateral frontopolar, dorsolateral, ventrolateral, and orbitofrontal PFC	1. In EO condition, OA with MCI displayed significantly higher HbO_2_ levels in left orbitofrontal cortex than healthy OA during standing on foam surface. 2. In the EC condition, OA with MCI exhibited significantly higher HbO_2_ levels than healthy OA across all PFC regions.	N/A

PFC, prefrontal cortex; OA, older adults; MA, middle-aged adults; YA, young adults; MCI, mild cognitive impairment; LF, low fear of falling; HF, high fear of falling; PSD, power spectral density; SMC, sensorimotor cortex; PMC, premotor cortex; SFG, dorsolateral superior frontal gyrus; SFGmedial, medial superior frontal gyrus; MFG, middle frontal gyrus; PreCG, precentral gyrus; PoCG, postcentral gyrus; MTG, middle temporal gyrus; SSC, somatosensory cortex; FEF, frontal eye fields; EO, eyes open; MSRS-C, Movement Specific Reinvestment Scale; FES-I, fall efficacy scale-international; SOT, Sensory Organization Test; MCT, Motor Control Test; ADT, Adaptation Test; SEO, eyes open on stable surface; SEC, eyes closed on stable surface; UEC, eyes closed on unstable surface; PF, postural-focus; SF, suprapostural-focus; HbO_2_, oxygenated hemoglobin. In the international 10–20 system, T3 and T4 correspond to T7 and T8, respectively, in the modified 10–20 system.

Thirteen studies (35.1%) used fNIRS. All fNIRS studies used HbO_2_ as an outcome measure, with 7 studies (Lin et al., 2017; Rosso et al., 2017; Marusic et al., 2019; Hinderaker et al., 2020; Rond et al., 2021; Lehmann et al., 2022) also reporting HHb. All but one study (Lin et al., 2017) measured cortical activation in the prefrontal cortex (PFC) region, with the dorsolateral PFC (*n* = 6) (Teo et al., 2018; Hinderaker et al., 2020; Xu et al., 2024a; Xu et al., 2024b; Pan and Tang, 2025; Pan and Zhang, 2025) assessed most frequently. A few fNIRS studies also measured (1) motor areas [supplementary motor area (SMA), premotor cortex] (Rosso et al., 2017; Hinderaker et al., 2020; Rond et al., 2021), (2) sensorimotor regions (including sensorimotor cortex, precentral gyri, postcentral gyri, somatosensory cortex) (Rond et al., 2021; Lehmann et al., 2022), (3) temporal and temporoparietal regions (middle temporal gyrus, temporoparietal) (Rosso et al., 2017; Hinderaker et al., 2020; Lehmann et al., 2022), and (4) occipital regions (Lin et al., 2017).

Several types of balance equipment were used to assess postural sway in included studies. Force plates were most frequently employed in the included studies to calculate various center of pressure (CoP) measures, such as CoP displacement, path length, and sway velocity, as well as estimated center of mass (CoM) projections (*n* = 14) (Ozdemir et al., 2016; Ozdemir et al., 2018; Bohle et al., 2019; Chan et al., 2019; Marusic et al., 2019; Hinderaker et al., 2020; George et al., 2021; Lehmann et al., 2022; Li et al., 2022; Xu et al., 2024a; Xu et al., 2024b; Pan and Tang, 2025; Pan and Zhang, 2025; Wang et al., 2025). Dynamic posturography (*n* = 5) (Rosso et al., 2017; Teo et al., 2018; Lin et al., 2021; Hu et al., 2023; Kan et al., 2025), inclinometer (*n* = 4) (Chen et al., 2021; Tsai et al., 2022; Chen et al., 2024; Chen et al., 2025), 3D motion capture (*n* = 4) (Chow et al., 2018; Malcolm et al., 2021; Payne et al., 2021; Rond et al., 2021), and inertial sensors (*n* = 2) (Rubega et al., 2021; Kahya et al., 2022) were also used to assess various standardized instrumented balance outcomes (e.g., Equilibrium Score) and CoM measures (e.g., CoM postural sway, error).

### Behavioral findings after sensory, dual-tasking, and mechanical challenges

Regardless of the measures used to assess postural sway and balance performance, we consistently observed greater postural sway in OA than in YA during sensory perturbation balance tasks (see Table 1). The magnitude of the difference in postural sway between OA and YA increased as task demands rose, indicating that sensory perturbation worsened postural stability more in OA than in YA. Similarly, within-group analyses in OA showed that visual, somatosensory, or combined perturbations resulted in greater postural sway compared with sensory-intact conditions (e.g., eyes open and comfortable stance).

Findings from studies involving dual-task conditions were inconsistent. Specifically, eight studies (Ozdemir et al., 2016; Huang et al., 2017; Bohle et al., 2019; Yu et al., 2019; Rond et al., 2021; Rubega et al., 2021; Xu et al., 2024a; Pan and Tang, 2025) revealed significant differences in postural sway between OA and YA, whereas five studies (Rosso et al., 2017; Marusic et al., 2019; Li et al., 2022; Kan et al., 2025; Pan and Zhang, 2025) did not. Interestingly, five of the eight studies that reported significant differences used EEG, whereas four of the five studies that reported null findings used fNIRS. Although studies reporting both significant and null findings used similar motor tasks, those reporting significant differences tended to employ more cognitively demanding tasks, such as visual-auditory (Bohle et al., 2019), force-matching (Huang et al., 2017; Yu et al., 2019), visual oddball (Rubega et al., 2021), and two-back tasks (Ozdemir et al., 2016). In contrast, studies reporting no significant group differences more often used simpler motor and cognitive tasks, such as holding a tray (Li et al., 2022) and performing serial subtraction tasks (Marusic et al., 2019; Pan and Zhang, 2025), respectively. Nevertheless, we found consistent patterns that cognitive performance during dual-tasking conditions was worse in OA, and the magnitude of this difference increased with greater task difficulty.

Similarly, Hu et al. (2023) indicated greater postural sway in OA and in YA during balance tasks with mechanical challenges. Palmer et al. (2021) reported a significant and strong correlation between dual-task interference, measured using the Timed Up and Go with serial subtraction by 3 s, and reactive step threshold. However, no associations were observed between general clinical balance function, as measured by the miniBEST, and either dual-task interference or reactive step threshold. Payne et al. (2021) reported that poorer cognitive set shifting ability was significantly associated with stiffer balance responses to perturbations.

### Age-related sensory perturbation on cortical activity

Table 3 summarizes age-related differences in regional spectral activity, functional connectivity, PAC, oxygenated hemoglobin levels, and associations between cortical activity and balance parameters during sensory perturbation conditions.

**TABLE 3:** A summary of age-related cortical modulation during sensory perturbation.

Experimental context/modulation	Included studies (N)	Effect direction	Main synthesis
1. Regional spectral power			
Delta and theta activity	6	Within-group modulation: 4 ↑, 2 ↓ between-group modulation: 2 studies: YA > OA	OA showed increased delta and theta power, primarily over frontal regions, during sensory challenges, while YA demonstrated significantly greater cortical modulation with increased task demands.
Alpha and beta activity	6	Within-group modulation: 4 ↑, 2 ↓ between-group modulation: 2 studies: YA > OA	1. OA showed increased alpha and/or beta power across frontal, central, parietal, and occipital regions. 2. OA exhibited higher baseline beta power than YA, but showed blunted cortical modulation compared to YA with increased task demands.
Gamma activity	3	Within-group modulation: 2 ↑ between-group modulation: 1 study: YA > OA	Sensory perturbations increased gamma power over frontal and central regions in OA, though YA showed greater cortical modulation.
2. Functional connectivity			
Phase-lag index (PLI)	2	2 ↑	Visual perturbations altered alpha-band functional connectivity in OA, redistributing connectivity across frontoparietal, occipital, visuospatial, and motor networks during balance tasks.
Coherence	4	Within-group modulation: 1 ↑ (T3–Fz), 2 ↔ (T4–Fz) between-group modulation: 1 study: YA > OA	OA exhibited less adaptable connectivity patterns across balance conditions, whereas YA showed greater sensory-dependent reorganization within mu- and alpha-band visual, somatosensory, and motor networks.
3. Phase-amplitude coupling (PAC)			
PAC	2	Within-group modulation: 1 ↑ between-group modulation: 1 study: YA > OA in theta- and alpha-band, OA > YA in beta-band	1. Visual interference increased theta-, alpha-, and beta-band PAC in OA across frontal, sensorimotor, and parietal regions. 2. During real-time visual feedback, OA compared to YA showed lower theta- and alpha-band PAC, but greater beta-band PAC over C3.
4. Oxygenated hemoglobin (HbO2)			
HbO_2_ level	6	Between-group modulation: 5 studies: OA > YA/MA 1 study: OA with MCI > healthy OA	HbO_2_ level increased with increased task difficulty in healthy OA and OA with MCI compared to control groups.
5. Associations between cortical activity and postural sway			
OA vs. YA	2	2 studies: YA > OA	OA showed weaker or absent associations between cortical activity and postural sway, whereas greater beta-band activity was associated with increased postural sway in YA.

↑, increased cortical modulation; ↓, decreased cortical modulation; ↔, null finding; OA, older adults; YA, younger adults; MA, middle-aged adults; MCI, mild cognitive impairment.

### Regional spectral activity

OA consistently showed increased delta and theta power in frontal regions and SMA in response to visual and somatosensory manipulations, although the magnitude of these increases was greater in YA. Specifically, two studies showed increased theta power in response to visual interference compared to the eyes open condition, particularly over Fz and Oz (Chang et al., 2016), as well as fronto-motor (Fp1/2, F7/8, F3/4, Fz, FT7/8, FC3/4, FCz, T3/4, C3/4, Cz) and parietal regions (P3, CP3) (Chen et al., 2025). Similarly, studies involving misleading somatosensory input or a reduced base of support showed elevated delta and theta activity in OA, primarily over frontal and supplementary motor areas (Ozdemir et al., 2016; Malcolm et al., 2021; Rubega et al., 2021). In contrast, two studies (Malcolm et al., 2021; Tsai et al., 2022) showed a reduction in theta power over frontal (Fz), sensorimotor areas (FC3, C3, CP3), and left precuneus cluster after visual interference. The between-group analyses indicated that YA showed greater increases in delta and theta power than OA when narrowing their base of support from a comfortable stance to a tandem stance (Malcolm et al., 2021) and when standing on a sway-referenced platform (Ozdemir et al., 2016).

Among the six studies involving sensory perturbations (Chang et al., 2016; Ozdemir et al., 2016; Lin et al., 2021; Malcolm et al., 2021; Tsai et al., 2022; Hu et al., 2023), four reported increased alpha and/or beta power in OA, with greater activity observed across frontal, central, parietal, and occipital regions (Chang et al., 2016; Ozdemir et al., 2016; Lin et al., 2021; Tsai et al., 2022). In contrast, Malcolm et al. (2021) indicated that visual interference significantly reduced alpha and beta power in SMA and left precuneus in OA. Hu et al. (2023) also reported that OA exhibited lower relative beta power during eyes-closed conditions on a sway platform compared to eyes open on a firm surface. Notably, between-group analyses showed that OA exhibited significantly higher beta or fast beta power than YA at midline electrodes (Fz, Cz, Pz) (Hu et al., 2023) and across bilateral temporal, parietal, and occipital regions (Lin et al., 2021). However, both studies indicated that YA demonstrated greater task-dependent modulation (i.e., dynamic increases or decreases in alpha and/or beta power across task conditions) than OA in response to increased task demands, whereas OA exhibited blunted EEG responses to sensory manipulations.

For gamma power, Chang et al. (2016) showed its increased activity during the visual interference condition at Fz and Oz. Similarly, gamma power significantly increased from standing on a firm surface with eyes open to standing on a sway platform condition with eyes closed, with OA exhibiting significantly higher gamma power in the central-parietal (CP3, CP1, CPz, CP2, CP4), central (C3, C1, Cz, C2, C4), and frontal (F3, F1, Fz, F2, F4) cortices compared to YA (Ozdemir et al., 2018). However, Malcolm et al. (2021) reported that YA but not OA exhibited increased gamma power during tandem stance compared to comfortable stance.

### Functional connectivity

Two studies employed Phase-Lag Index (PLI), a measure of functional connectivity by estimating phase synchronization between different EEG signals, during visual perturbations. Both studies indicated that connectivity changes were most prominent in the alpha band, particularly across posterior frontoparietal-occipital or visuospatial networks, suggesting that visual disruption redistributes functional connectivity in OA during balance tasks. Specifically, Tsai et al. (2022) compared PLI when OA stood on the stabilometer with and without intermittent visual interference (i.e., stroboscopic vision). The authors indicated that intermittent visual interference significantly increased alpha-band PLI (i.e., enhanced functional connectivity) over the right-hemisphere visual dorsal and frontal–occipital networks, and also altered beta-band PLI, whereas theta-band effects were marginal. Similarly, Chen et al. (2021) compared PLI while OA stood on a foam surface with eyes open and eyes closed. Relative to the eyes-open condition, the eyes-closed condition significantly reduced fronto-tempo-motor connectivity and enhanced fronto-parietal-occipital connectivity, with the most prominent changes observed in the alpha band.

Four studies examined coherence as a measure of functional connectivity. Wang et al. (2025) found stronger mu-band (8–13 Hz) coherence across cortical regions in YA and OA under both visually congruent and incongruent conditions. Under visually congruent conditions, YA exhibited stronger coherence within visual integration networks. However, under the visual-conflict condition where visual information was incongruent with self-motion, YA exhibited increased mu-band coherence within somatosensory and motor integration networks, suggesting a shift from reliance on visual information toward greater reliance on motor-centered information to maintain balance. Conversely, OA showed more similar connectivity patterns across visually congruent and incongruent conditions, suggesting reduced adaptability of neural connectivity to changes in the sensory environment. Among three studies (Chow et al., 2018; Chu and Wong, 2019; Li et al., 2022) specifically examining T3–Fz and/or T4–Fz coherence (international 10–20 system), only Chu and Wong (2019) showed significantly increased T3–Fz fast alpha (10–12 Hz) coherence as the base of support decreased. None of the studies showed significant changes in T4–Fz coherence with different base of support positions (Chu and Wong, 2019; Li et al., 2022).

Chang et al. (2016) performed Pearson’s correlation coefficient to investigate the cortical relationship between postural-related cortex regions. Of note, during the platform recovery phase with virtual reality interference, the low fall-risk group exhibited significant positive correlations between Fz and Cz, and between Fz and Pz across the theta, alpha, beta, and gamma bands (*r* = 0.538–0.576), between Fz and Oz in the alpha, beta, and gamma bands (*r* = 0.529–0.543), and between Pz and Oz across all theta, alpha, beta, and gamma bands (*r* = 0.858), whereas the high fall-risk group showed no significant correlations of power spectrum density (Chang et al., 2016).

### Phase-amplitude coupling (PAC)

Two studies assessed PAC, which quantifies the relationship between the phase of low-frequency postural fluctuations and the amplitude of EEG oscillations, in response to sensory challenges during standing balance, and both studies reported greater beta band PAC in sensorimotor regions. Specifically, Chen et al. (2025) reported increased theta- and alpha-band PAC in fronto-central regions (Fp1, Fp2, F7, F3, Fz, F4, F8, FT7, FC3, FCz, FC4, FT8, T3, C3, Cz, C4) during intermittent visual interference (i.e., stroboscopic vision) compared to full vision, suggesting increased temporal coordination between postural fluctuations and cortical oscillations under greater balance demands. The authors also observed significantly increased beta-band PAC in frontal (Fp1, Fp2, F3, Fz, F4, F8) and sensorimotor (FC3, FCz, FC4, T3, C3, C4, CP3) areas, potentially reflecting altered cortico-postural interactions related to open-loop postural control during stabilometer stance with intermittent visual interference. Another study by Chen et al. (2024) compared PAC between YA and OA while performing stabilometer stance with real-time visual feedback from a computer display showing the platform’s movement trajectory and a horizontal target representing ground level. The authors indicated that OA compared to YA exhibited lower theta-band PAC in FCz and bilateral temporal-parietal-occipital regions, and lower alpha-band PAC in posterior regions, but greater beta-band PAC over C3. The authors indicated that weaker theta-band PAC in OA may reflect an age-related reduction in error detection and the planning of corrective postural responses based on visual feedback. Lower alpha-band PAC in posterior regions may reflect weaker coupling between visuospatial cortical processing and postural fluctuations under increased visual processing demands. In contrast, stronger sensorimotor beta-band PAC may indicate a more rigid and less adaptable postural control strategy during unstable stance.

### Oxygenated hemoglobin

During sensory perturbations, we observed consistent findings of increased HbO_2_ levels in response to greater task difficulty, and HbO_2_ levels were greater in OA than in YA or MA (Lin et al., 2017; Teo et al., 2018; Hinderaker et al., 2020; George et al., 2021; Lehmann et al., 2022). Similarly, Xu et al. (2024b) compared cortical activation differences between OA with and without MCI, indicating that OA with MCI exhibited significantly higher HbO_2_ levels than healthy OA across all PFC regions across all conditions than those without MCI.

### Associations between cortical activity and balance parameters during sensory-related standing balance tasks

We found relatively consistent findings that relationships between cortical activity and postural sway decrease in OA (Malcolm et al., 2021; Hu et al., 2023). For example, during the sensory organization test, no correlations were found between postural sway and beta band in OA, whereas higher beta power was significantly and moderately correlated with lower equilibrium scores (i.e., greater postural sway; ρ = -0.44, *p* < 0.001) in YA at Pz (Hu et al., 2023). Similarly, significant correlations were observed between mediolateral sway and beta power in the right frontal cluster (*r* = 0.67, *p* = 0.048) and the left parietal cluster (*r* = 0.66, *p* = 0.036) when task demands increased in YA, while no such relationships were found in OA (Malcolm et al., 2021).

Across studies, the relationship between cortical activity and postural sway appeared to be highly task dependent, becoming stronger as sensory or balance demands increased, although the direction of these associations varied by cortical region and neural measure. For example, Ozdemir et al. (2018) reported that higher delta power was significantly and positively correlated with time to boundary (i.e., lower postural sway; *r* = 0.25–0.33, *p* < 0.05) during challenging balance tasks (e.g., eyes closed and/or standing on unstable surface) in OA, whereas little to no relationships were found across all frequency bands under relatively stable balance conditions. Likewise, larger increases in mid-frontal theta power in response to visual interference were positively correlated with RMS of angular fluctuations (i.e., greater postural sway; *r* = 0.36, *p* = 0.04) (Tsai et al., 2022). Similarly, Teo et al. (2018) found that higher PFC HbO_2_ was associated with higher equilibrium scores (i.e., better balance performance) only during more sensory-demanding sensory organization test conditions, whereas no such relationship was observed in easier conditions. Wang et al. (2025) further showed that cortical–posture relationships in OA depended on sensory context, such that motor cortex efficiency was associated with greater postural sway area under sensory-congruent conditions, whereas greater visual cortex efficiency was associated with lower postural sway area only under sensory-conflict conditions. In contrast, Chen et al. (2021) reported significant correlations between various measures of stabilogram diffusion analysis, which characterize the shift in feedback and feedforward controls of the CoP, and delta, theta, and beta activity in the eyes open condition, whereas no such associations emerged in the eyes closed condition.

### Age-related effects of dual-tasking on cortical activity

Table 4 summarizes age-related differences in regional spectral activity, functional connectivity, ERPs, oxygenated hemoglobin levels, and associations between cortical activity and balance parameters during dual-task conditions.

**TABLE 4:** A summary of age-related cortical modulation during dual-tasking.

Experimental context/modulation	Included studies (N)	Effect direction	Main synthesis
1. Regional spectral power			
Delta and theta activity	4	Within-group modulation: 4 ↑ between-group modulation: 2 studies: YA > OA 1 study: OA > YA	1. OA consistently increased delta- and theta-band activity from ST to DT over the frontal, central, and posterior regions. 2. We observed a tendency toward greater delta- and theta-band modulation in YA than in OA during dual-task conditions.
Alpha activity	4	Within-group modulation: 2 ↑, 2 ↓ between-group modulation: 3 studies: YA > OA	Direction of alpha activity was mixed, whereas YA compared to OA consistently exhibited greater alpha modulation during DT.
Beta activity	4	Within-group modulation: 1 ↑, 3 ↔ Between-group modulation: 2 studies: no differences	Beta activity remained largely unmodulated between ST and DT in OA, with no significant differences observed between YA and OA.
Gamma activity	2	Within-group modulation: 2 ↑ between-group modulation: 2 studies: OA > YA	DT increased frontal and centroparietal gamma power significantly more in OA than in YA.
2. Functional connectivity			
Synchronization likelihood method	1	Between-group modulation: 1 ↑	Increasing postural load during DT enhanced fronto-sensorimotor connectivity in OA compared to YA.
Coherence	1	Within-group modulation: 1 ↔ (T3–Fz, T4–Fz)	No significant differences were observed between ST and DT in OA.
3. Event-related potentials (ERPs)			
N1 and P2	2	Between-group modulation: N1: mixed findings across 2 studies P2: no differences in 1 study	Inconsistent age-related differences were observed in preparatory ERPs, particularly N1 amplitude, while P2 responses showed either attenuation in OA or no significant age-related differences.
4. Oxygenated hemoglobin (HbO2)			
HbO_2_ level (between-group)	5	Between-group modulation: 4 studies: OA > YA 1 study: no difference	OA exhibited significantly greater HbO_2_ activation in PFC, sensorimotor, and temporal regions during DT compared to YA.
HbO_2_ level (within-group)	6	Within-group modulation: 3 ↑, 3 ↓	Relative changes in PFC and sensorimotor HbO_2_ levels from ST to DT were mixed.
5. Associations between cortical activity and postural sway			
ST	4	Within-group: 4 ↑	Significant correlations were observed between HbO_2_ levels and postural sway measures in OA, although the directions of these associations were mixed.
DT	4	Within-group: 4 ↔, 1 ↑ (OA with MCI)	Correlations between PFC HbO_2_ levels and postural sway measures during DT were not significant in OA.

↑, increased cortical modulation; ↓, decreased cortical modulation; ↔, null finding; OA, older adults; YA, younger adults; MCI, mild cognitive impairment; ST, single-task; DT, dual-task; PFC, prefrontal cortex.

### Regional spectral activity

Dual-task standing generally increased low-frequency cortical activity in OA. All EEG studies (Ozdemir et al., 2016; Bohle et al., 2019; Rubega et al., 2021; Kahya et al., 2022) reported that dual-task standing, with and without visual and somatosensory manipulations, significantly increased delta and theta power over the frontal, central, and posterior cortical regions compared to single-task conditions in OA, suggesting that the delta activity is more responsive to postural challenges and the theta activity to cognitive load during dual-tasking (Ozdemir et al., 2016; Bohle et al., 2019). However, two studies indicated that YA exhibited higher delta and theta activities than OA in the mid-anterior (Fz) and central-frontal (FC5, FC3, FC1, FC2, FC4, FC6) areas during dual-tasking balance tasks (Ozdemir et al., 2016; Bohle et al., 2019), respectively, whereas one study (Rubega et al., 2021) found greater frontal and occipital theta power in OA than in YA.

During dual-task balance tasks, cortical activity patterns were mixed in the alpha band and remained largely unchanged in the beta band relative to single-task balance conditions in both OA and YA. However, across studies, YA generally exhibited greater alpha-band modulation than OA. Two studies reported a reduction in the alpha activity from single-task conditions to dual-task conditions in the central-left (C3, CP1, CP5, T7), central-right (C4, T8, CP2, CP6), and posterior-left (P7, P3, PO1, PO3) regions (Bohle et al., 2019; Kahya et al., 2022), with more pronounced decreases in YA (Bohle et al., 2019). Ozdemir et al. (2016) reported that increasing cognitive difficulty from one-back to two-back tasks increased alpha power over parietal and occipital cortices in both YA and OA, while more pronounced increases in alpha power were shown in YA over occipital regions. Rubega et al. (2021) compared alpha and beta power during dual-tasking on a firm surface and on a balance board in OA, indicating increased alpha and beta power over sensorimotor regions during the balance board condition. Of note, most studies found no significant beta activity differences between single- and dual-tasks (Ozdemir et al., 2016; Bohle et al., 2019; Kahya et al., 2022) and between YA and OA (Ozdemir et al., 2016; Bohle et al., 2019). For the gamma power, two studies (Ozdemir et al., 2016; Rubega et al., 2021) showed that gamma power over frontal and central-parietal (CP3, CP1, CPz, CP2, CP4) regions increased during dual-task relative to single-task conditions, and this increase was significantly greater in OA than in YA.

### Functional connectivity

Functional connectivity findings suggest that dual-tasking increases frontal network engagement with age-related differences in posterior network reorganization. Huang et al. (2017) assessed functional connectivity of ERP using the synchronization likelihood (SL) method. The authors reported that increasing postural load during the force-matching task increased global and fronto-sensorimotor functional connectivity in both YA and OA, and OA showed overall stronger fronto-sensorimotor connectivity than YA. In contrast, YA showed reduced parietal-occipital connectivity and decreased left temporal–parietal-occipital coupling under greater postural load, whereas OA showed increased parietal–occipital connectivity and enhanced right prefrontal connectivity under the same condition. Li et al. (2022) who examined T3–Fz and T4–Fz coherence between single- and dual-task tandem stance, reported no significant changes between the two tasks.

### Event-related potentials (ERPs)

Two studies assessed preparatory ERP responses during force-matching balance tasks, suggesting age-related differences in early and late cortical processing, although the direction of effects varied across studies. Yu et al. (2019) indicated that OA showed significantly greater N1 peaks compared to YA in the frontal and sensorimotor-parietal regions, while OA showed attenuated P2 peaks compared to YA across posture-focus and supra-posture-focus conditions. On the other hand, Huang et al. (2017) found larger N1 amplitudes in YA than in OA over frontal and sensorimotor regions, no group differences in parietal-occipital regions, and larger P2 amplitudes on the dynamic than firm surface in both groups, with no significant age-related differences.

### Oxygenated hemoglobin

fNIRS studies consistently showed greater and more widespread cortical activation in OA during dual-task balance compared to YA. OA showed greater and widespread HbO_2_ activation over the PFC and sensorimotor cortical areas (George et al., 2021; Rond et al., 2021; Pan and Tang, 2025), as well as the temporal gyrus and supramarginal gyrus (Rosso et al., 2017), during dual-task conditions compared to YA. Similarly, OA with MCI showed significantly greater HbO_2_ levels than those without MCI during both single- and dual-task balance tasks (Xu et al., 2024a). Only Marusic et al. (2019) did not find age-related differences in PFC activity between OA and YA.

However, task-related HbO_2_ changes from single-task to dual-task conditions were mixed. Three studies revealed heightened cortical activation during dual-task conditions compared to single-task conditions over the PFC and sensorimotor areas (Marusic et al., 2019; Kan et al., 2025; Pan and Zhang, 2025), while other studies reported decreased HbO_2_ responses over the same areas during dual-task conditions relative to single-task conditions (Rosso et al., 2017; George et al., 2021; Rond et al., 2021).

### Associations between cortical activity and balance parameters during dual-tasking standing tasks

Only one EEG study examined cortical and postural relationships (Kahya et al., 2022). The authors reported that a greater increase in theta/beta power ratio from single-task standing to dual-task standing was associated with a greater dual-task cost to postural sway area over the anterior-left (*r* = 0.52, *p* < 0.01; F7, Fp1, F3, FC1, FC5, AF3), central-right (*r* = 0.53, *p* < 0.01; C4, T8, CP2, CP6), and posterior-left (*r* = 0.45, *p* < 0.01; P7, P3, PO1, PO3) regions, indicating that greater attentional control-related cortical change was indicative of worse balance outcomes in OA. They also found greater alpha power in the anterior-right (*r* = 0.52, *p* < 0.01; F4, Fp2, F8, FC2, FC6, AF4) and central-right (*r* = 0.48, *p* < 0.01) regions was correlated with greater postural sway velocity during dual-tasking, suggesting that altered oscillatory activity is related to postural control.

Overall, fNIRS studies showed that the relationships between PFC HbO_2_ levels and balance performance varied across task conditions, cognitive status, and age groups, with associations generally more evident during single-task conditions in OA. All fNIRS studies examining the relationships reported no correlations between PFC HbO_2_ levels and postural sway measures during dual-task conditions in healthy OA (Rond et al., 2021; Xu et al., 2024a; Pan and Zhang, 2025). However, in OA with MCI, PFC HbO_2_ levels were positively correlated with RMS displacement in the anteroposterior direction (*r* = 0.45, *p* = 0.04) and the 95% confidence ellipse area (*r* = 0.49, *p* = 0.04) (Xu et al., 2024a). During single-task conditions, two studies indicated that greater PFC HbO_2_ levels were correlated with greater overall sway path (Xu et al., 2024a) and CoP oscillations at 0–0.1 Hz (Pan and Zhang, 2025), whereas Rond et al. (2021) reported that higher PFC HbO_2_ levels were positively correlated with the number of wasps hit during the game (*r* = 0.47, *p* = 0.03), indicating better balance performance. In contrast, among OA with MCI, higher PFC HbO_2_ levels were negatively correlated with RMS displacement in the anteroposterior direction (*r* = -0.47, *p* = 0.03) and maximum CoP displacement in the anteroposterior direction (*r* = -0.47, *p* = 0.03). Importantly, unlike OA, YA showed significantly positive correlations between PFC levels and CoP oscillations at 0–0.1 Hz and at 0.1–0.5 Hz in both single- and dual-task conditions (Pan and Zhang, 2025).

### Age-related effects of mechanical challenges on cortical activity

#### Regional spectral activity

Studies consistently reported that mechanical perturbations led to significantly higher beta power and greater RMS amplitude in OA compared to YA over the frontal (F3, Fz, F4) and central (C3, Cz, C4) regions (Saadat et al., 2021; Hu et al., 2023; Wang et al., 2023).

#### Functional connectivity

One study demonstrated higher alpha coherence in the F3–P3 and F4–P4 regions, as well as higher beta coherence in the F4–P4 region during unpredictable mechanical perturbations in OA (Saadat et al., 2021), whereas another reported that balance perturbations did not significantly alter CPz–Cz (somatosensory–motor) or AFz–Cz (prefrontal–motor) beta coherence in OA (Palmer et al., 2021).

#### Event-related potentials (ERPs)

Ozdemir et al. (2018) reported that OA had significantly longer P1 and N1 latencies than YA, primarily over the central and central-parietal cortices. YA also showed significantly greater N1 amplitude than OA over the central cortices.

#### Associations between cortical activity and balance parameters during mechanical challenges

Studies offering mechanical challenges revealed relatively consistent findings of significant relationships between beta power and balance parameters. Hu et al. (2023) showed that relative beta power was positively correlated with average latency (i.e., slower reaction to perturbation) at Fz (ρ = 0.299, *p* < 0.05) and Cz (ρ = 0.315, *p* < 0.05) in OA, but not in YA, during the Motor Control Test, where participants completed six conditions comprising three forward and three backward translations at small (2.8°/s), medium (6.0°/s), and large (8.0°/s) velocities, scaled to each participant’s height. However, relative beta power was not significantly related to sway energy in the Adaptation Test paradigms, which contain two different conditions, including toes-up and toes-down, with each involving an 8° platform rotation at a rate of 20°/s. Palmer et al. (2021) reported that higher perturbation-evoked beta power during the late-phase of balance reactions (300–500 ms) was significantly correlated with low miniBEST score (i.e., worse balance; *r* = –0.56, *p* = 0.04), with no relationships observed during overall (100–500 ms) and early- (100–300 ms) phase. The authors also revealed no relationship between miniBEST score and either perturbation-evoked CPz–Cz (somatosensory–motor) or AFz–Cz (prefrontal–motor) beta coherence. Payne et al. (2021) reported that the miniBEST score was not significantly related to the peak amplitude of cortical N1 responses (100–200 ms after onset) at Cz during unpredictable mechanical perturbations.

## Discussion

There has been a growing interest in age-related cortical mechanisms of postural control over the past decade. To our knowledge, this is the first systematic review to comprehensively synthesize cortical activity and its relationship with postural control during standing balance tasks involving sensory, cognitive, and mechanical challenges in OA. After identifying distinct cortical activity patterns across the power spectrum of frequency bands, functional connectivity, ERPs, and HbO_2_ responses during varying balance tasks, we found several notable patterns that may help understand the greater postural sway seen in OA compared to YA. Overall, OA appeared to exhibit less task-dependent cortical modulation and weaker associations between cortical activity and postural sway than YA during challenging balance conditions. These patterns may be consistent with age-related differences in sensory reweighting and reduced neural efficiency. Similarly, ERP findings of prolonged latencies and reduced amplitudes following mechanical perturbation may further reflect slower sensory processing in OA. At the same time, other findings showed greater cortical activation under certain task conditions in OA, which may indicate compensatory recruitment.

EEG frequency-domain findings suggest that OA may show reduced task-dependent cortical modulation as sensory and cognitive demands increase. During sensory-challenging balance conditions, blunted modulation of delta, theta, alpha, and beta activity relative to sensory-intact conditions may reflect less flexible sensory reweighting, particularly across sensorimotor and sensory association regions in the parietal, temporal, and occipital cortices (Ozdemir et al., 2016; Ozdemir et al., 2018; Lin et al., 2021; Malcolm et al., 2021). Such reduced adaptability to altered or conflicting sensory input may partly underlie the greater postural sway observed in OA under sensory challenge (Ozdemir et al., 2016; Ozdemir et al., 2018; Lin et al., 2021; Malcolm et al., 2021). A similar pattern was evident during dual-task conditions, in which OA generally showed smaller task-related changes in delta, theta, and alpha activity than YA. Given that delta and theta power are shown to be sensitive to changes in postural demands and cognitive efforts, respectively (Ozdemir et al., 2016; Bohle et al., 2019; Peskar et al., 2025), these attenuated responses in OA compared to YA may indicate a reduced capacity to dynamically allocate attentional resources, detect errors, and adapt to increased task demands. Previous studies have linked reduced alpha-band activity in sensorimotor and posterior regions to increased cortical activation under greater postural demands (Bohle et al., 2019; Malcolm et al., 2021). The reduced alpha activity may also reflect a release from cortical inhibition, allowing task-relevant regions to become more responsive to incoming sensory and cognitive information (Klimesch, 2012; Giovanni et al., 2017). Thus, greater alpha reduction in YA relative to OA likely reflects a more effective neural disinhibition mechanism, supporting the simultaneous processing of postural control and secondary cognitive loads.

The weaker association between cortical activity and postural sway in OA and high fall-risk groups, relative to YA and lower-risk groups, may provide additional insight into age-related balance impairment. Rather than reflecting efficient cortical adaptation, greater cortical activation in OA during less demanding tasks may indicate increased effort that is not functionally sufficient to preserve postural stability. This interpretation is broadly consistent with the Compensation-Related Utilization of Neural Circuits Hypothesis (CRUNCH), whereby OA may recruit greater neural resources during lower-demand tasks, leaving less reserve available as task difficulty increases (Reuter-Lorenz and Cappell, 2008). Under this framework, weaker and/or absent associations between cortical activity and postural control in OA may reflect limited flexibility of cortical modulation, such that increased cortical recruitment may not reliably translate into better balance performance (Zarahn et al., 2007; Bernard and Seidler, 2012). Together, these findings suggest that age-related increases in cortical activation should not be interpreted as uniformly beneficial, but rather in relation to whether they are effectively coupled to postural outcomes.

Mechanical perturbation studies further suggest that OA may rely more on higher-order cortical processing during reactive postural control. Increased beta power following perturbation (Saadat et al., 2021; Tsai et al., 2022), together with region-specific beta coherence patterns (Palmer et al., 2021; Saadat et al., 2021), indicates that OA may depend more on frontoparietal integrative networks than on direct sensorimotor coupling (Lenz et al., 2012; Palmer et al., 2021). This is supported by findings showing significant beta coherence observed between frontal (F3, F4) and parietal (P3, P4) areas (Saadat et al., 2021), but not between somatosensory (CPz) and motor (Cz) areas (Palmer et al., 2021). In parallel, prolonged P1 and N1 latencies in OA suggest delayed sensory processing and attentional allocation following perturbation onset (Ozdemir et al., 2018). Taken together, these findings may be consistent with reduced automaticity of postural control in aging, with greater reliance on slower, higher-order cortical mechanisms during balance recovery. However, these interpretations remain inferential and should be confirmed in studies that directly assess the proposed mechanisms.

Despite evidence of reduced cortical adaptability, several studies also reported increased cortical activity in OA during challenging balance tasks, a pattern that may be consistent with preserved or compensatory recruitment (Ozdemir et al., 2016; Ozdemir et al., 2018; Kahya et al., 2022; Tsai et al., 2022). Increases in delta-, theta-, and gamma-band activity during sensory-challenging and dual-task conditions, particularly over frontal, sensorimotor, and parietal regions, may reflect greater task-related recruitment of neural resources to support balance when demands increase in OA. Notably, significant associations between delta power, theta power, the theta/beta ratio, and postural sway indicate that these oscillatory responses remain functionally relevant under challenging conditions in OA (Ozdemir et al., 2018; Kahya et al., 2022; Tsai et al., 2022). Frontal and sensorimotor theta activity, in particular, has been linked to motor control, conflict monitoring, and sensory integration (Gramann et al., 2011; Hülsdünker et al., 2015; Eisma et al., 2021), whereas low-frequency oscillations more broadly may facilitate communication across distributed cortical networks (Babiloni et al., 2017). Similarly, increased gamma power during both sensory-challenging and dual-task conditions, particularly over frontal, central, and parietal regions, may be consistent with heightened attentional engagement (Ozdemir et al., 2016; Ozdemir et al., 2018). Taken together, increases in delta-, theta-, and gamma-band activity over frontal, sensorimotor, and parietal regions may represent neural correlates of increased postural demands in OA, although further studies are needed to clarify their functional significance and relationship with balance performance.

Alpha- and beta-band findings further suggest that cortical responses among OA are highly context dependent. During sensory perturbation, alpha and/or beta power generally increased across frontal, central, parietal, and occipital regions (Chang et al., 2016; Ozdemir et al., 2016; Lin et al., 2021; Tsai et al., 2022). Mechanistically, increased alpha-band activity may reflect greater inhibitory gating of task-irrelevant sensory information (Klimesch, 2012), which may reflect a top-down strategy to prioritize sensory processing, executive control, and sensorimotor integration (Chang et al., 2016; Lin et al., 2021; Tsai et al., 2022; Chen et al., 2025). In contrast, under dual-task conditions, alpha-band findings were inconsistent, whereas beta activity showed little change relative to single-task conditions (Ozdemir et al., 2016; Bohle et al., 2019; Kahya et al., 2022). These mixed findings may reflect heterogeneity in the cognitive tasks used across studies, which likely imposed different attentional, executive, and postural control demands. Accordingly, alpha and beta oscillations in OA may be better understood as markers of context-specific control processes rather than as reflecting a single age-related pattern across all balance conditions.

Functional connectivity findings suggest that age-related postural control may depend not only on the magnitude of cortical activation, but also on the organization of distributed networks. Under visual perturbation, connectivity tended to shift toward longer-range fronto-occipital networks (Chen et al., 2021; Tsai et al., 2022), which may reflect increased coordination between frontal regions and posterior visuospatial areas when visual input becomes unreliable (Peterson and Ferris, 2019). Increased alpha connectivity across these regions may further indicate suppression of unreliable visual information and greater reliance on alternative sensory inputs. Furthermore, during dual-task conditions with increased postural demands, OA showed strengthened fronto-sensorimotor and right prefrontal functional connectivity compared with YA, consistent with compensatory recruitment in response to reduced postural automaticity (Huang et al., 2017). However, despite the redistributed and/or strengthened functional connectivity in response to greater sensory and cognitive demands, significantly greater postural sway was observed. Moreover, the limited evidence linking functional connectivity to postural sway makes it unclear whether these network changes reflect effective adaptation or less efficient control strategies. These findings nevertheless highlight functional connectivity as a promising systems-level marker of postural control that warrants further investigation.

fNIRS studies showed relatively consistent increases in HbO_2_ during sensory manipulations, suggesting greater cortical involvement under altered sensory conditions. In contrast, findings from dual-task paradigms were less consistent, with several studies reporting reduced PFC HbO_2_ during dual-task relative to single-task conditions (Rosso et al., 2017; George et al., 2021; Rond et al., 2021). This pattern is consistent with a previous systematic review, which also noted reduced prefrontal HbO_2_ during dual-task walking in OA (Kahya et al., 2019). One possible explanation is that OA can increase prefrontal recruitment under lower task demands but show reduced responses once task difficulty exceeds available cortical capacity (Kahya et al., 2019). Under lower task demands, OA may recruit additional prefrontal resources to compensate for age-related declines in balance and cognitive processing, resulting in increased HbO_2_—a finding consistent with the CRUNCH framework. However, when task demands exceed their available processing capacity, they may reach a neural-resource ceiling and become unable to sustain or further increase cortical recruitment, leading to a plateau or reduction in HbO_2_ (Reuter-Lorenz and Cappell, 2008). However, this interpretation should be made cautiously because most fNIRS studies focused primarily on the PFC. Such limited spatial coverage makes it difficult to determine whether reduced HbO_2_ reflects failed recruitment, a redistribution of activity to other cortical regions, or alternative mechanisms such as sensory reweighting. Future studies using broader multi-channel coverage across sensorimotor, parietal, and temporal regions are needed to clarify whether age-related responses reflect compensatory prefrontal recruitment, capacity limits under high demand, or task-dependent reorganization of distributed postural-control networks.

Findings across the included EEG and fNIRS studies were generally consistent in showing that OA tended to exhibit less task-specific cortical modulation as task demands increased. This convergence is notable because the two modalities provide complementary information about cortical involvement in postural control. EEG offers high temporal resolution and can assess rapid changes in cortical oscillatory activity across multiple cortical regions, making it well-suited for examining dynamic balance responses and responses to sensory or mechanical perturbations (Palmer et al., 2021; Payne et al., 2021). However, EEG has relatively limited spatial resolution compared to magnetic resonance imaging and is relatively susceptible to movement and muscle artifacts (Herold et al., 2018). In contrast, fNIRS provides high spatial resolution of cortical hemodynamic activity and is relatively tolerant of movement, but its temporal resolution is limited by the delayed hemodynamic response (Herold et al., 2018). Moreover, most of the included fNIRS studies focused predominantly on the PFC, limiting the generalizability of their findings to other cortical regions closely involved in postural control. Given that cortical responses to mechanical perturbations have been relatively understudied in this review, EEG may be particularly suitable for future studies examining the rapid and distributed cortical dynamics of postural control in OA.

This review has several limitations. This study did not conduct a meta-analysis due to substantial heterogeneity in cortical outcome measures and insufficient data reporting across the included studies. Future research incorporating standardized behavioral and neurophysiological outcomes and more transparent reporting practices is recommended to enable a future meta-analysis further capable of extending our findings. Since this review primarily focused on cortical activities and their associations with postural sway, we did not attempt to analyze muscle activity measured by electromyography (EMG), which is another indirect indicator of neural activity underlying postural control. Future research examining the relationships between muscle and cortical activity during standing balance tasks may provide additional insights into the underlying mechanisms of postural control. The generalizability of our findings was limited to healthy OA, given that only two fNIRS studies (Xu et al., 2024a; Xu et al., 2024b) and one EEG study (Chang et al., 2016) in this review recruited OA with MCI and those at high risk of falling, respectively. Thus, greater efforts are needed to recruit specific aging subgroups, such as individuals with MCI, high fear of falling, or low multisensory integration, to better delineate neural mechanisms underlying postural control and to inform the development of targeted interventions for those at increased risk for falls. Furthermore, investigating how potential mediators and/or moderators (e.g., multisensory integration magnitude) influence balance-related cortical activity may provide mechanistic insight into age-related balance impairments. Unmeasured factors, including fear of falling, emotional arousal, vigilance, and reduced automaticity, may also contribute to age-related differences in cortical responses during balance tasks. Future studies that assess these factors are warranted to distinguish alternative explanations for the observed neural findings. Substantial heterogeneity also existed in signal preprocessing, frequency band definitions, artifact rejection strategies, and connectivity metrics, which likely contributed to inconsistent findings and limited cross-study comparability. Finally, the heterogeneity of balance measures across the included studies limits direct comparisons and should be considered when interpreting the relationships between cortical activity and postural sway.

## Conclusion

This systematic review advances current understanding of the cortical mechanisms underlying postural control in OA compared with YA by highlighting diminished task-dependent cortical modulation, weaker associations between cortical activity and postural sway, and delayed sensorimotor processing during sensory, cognitive, and mechanically challenging balance tasks. At the same time, evidence of preserved or increased cortical recruitment under some conditions suggests that age-related postural control reflects a complex interplay between reduced neural efficiency and compensatory engagement. These findings provide an important foundation for developing more standardized balance protocols and selecting more appropriate neural and behavioral outcomes in future studies. Greater methodological consistency may help improve comparability across studies and clarify age-related cortical mechanisms of postural control.

## Data Availability

The original contributions presented in the study are included in the article/Supplementary material, further inquiries can be directed to the corresponding author.

## References

[B1] Abou L. Peters J. Fritz N. E. Sosnoff J. J. Kratz A. L. (2022). Motor cognitive dual-task testing to predict future falls in multiple sclerosis: a systematic review. *Neurorehabil. Neural Repair* 36 757–769. 10.1177/15459683221131791 36320121

[B2] Adam C. E. Fitzpatrick A. L. Leary C. S. Ilango S. D. Phelan E. A. Semmens E. O. (2024). The impact of falls on activities of daily living in older adults: a retrospective cohort analysis. *PLoS One* 19:e0294017. 10.1371/journal.pone.0294017 38170712 PMC10763967

[B3] Ambrose A. F. Paul G. Hausdorff J. M. (2013). Risk factors for falls among older adults: a review of the literature. *Maturitas* 75 51–61. 10.1016/j.maturitas.2013.02.009 23523272

[B4] Babiloni C. Del Percio C. Lopez S. Di Gennaro G. Quarato P. P. Pavone L. et al. (2017). Frontal functional connectivity of electrocorticographic delta and theta rhythms during action execution versus action observation in humans. *Front. Behav. Neurosci.* 11:20. 10.3389/fnbeh.2017.00020 28223926 PMC5294389

[B5] Bae M. VanNostrand M. (2025). Cognition and measures of physical activity, mobility, and gait in individuals with multiple sclerosis: a systematic review. *Neurorehabil. Neural. Repair.* 39 559–577. 10.1177/15459683251335315 40317119

[B6] Bernard J. A. Seidler R. D. (2012). Evidence for motor cortex dedifferentiation in older adults. *Neurobiol. Aging* 33 1890–1899. 10.1016/j.neurobiolaging.2011.06.021 21813213 PMC3391352

[B7] Bohle H. Rimpel J. Schauenburg G. Gebel A. Stelzel C. Heinzel S. et al. (2019). Behavioral and neural correlates of cognitive-motor interference during multitasking in young and old adults. *Neural Plast.* 2019:9478656. 10.1155/2019/9478656 31582967 PMC6748191

[B8] Chan D. C. L. Wong T. W. L. Zhu F. F. Cheng Lam C. Young W. R. Capio C. M. et al. (2019). Investigating changes in real-time conscious postural processing by older adults during different stance positions using electroencephalography coherence. *Exp. Aging Res.* 45 410–423. 10.1080/0361073x.2019.1664450 31514583

[B9] Chang C.-J. Yang T.-F. Yang S.-W. Chern J.-S. (2016). Cortical modulation of motor control biofeedback among the elderly with high fall risk during a posture perturbation task with augmented reality. *Front. Aging Neurosci.* 8:80. 10.3389/fnagi.2016.00080 27199732 PMC4848299

[B10] Chen Y. C. Huang C. C. Zhao C. G. Hwang I. S. (2021). Visual effect on brain connectome that scales feedforward and feedback processes of aged postural system during unstable stance. *Front. Aging Neurosci.* 13:679412. 10.3389/fnagi.2021.679412 34366825 PMC8339373

[B11] Chen Y. C. Tsai Y. Y. Huang W. M. Zhao C. G. Hwang I. S. (2024). Age-related topological organization of phase-amplitude coupling between postural fluctuations and scalp EEG during unsteady stance. *IEEE Trans. Neural Syst. Rehabil. Eng.* 32 3231–3239. 10.1109/tnsre.2024.3451023 39196741

[B12] Chen Y. C. Tsai Y. Y. Lin Y. T. Hwang I. S. (2025). Enhancing anticipation control of the posture system in the elderly wearing stroboscopic glasses. *J. Neuroeng. Rehabil.* 22:104. 10.1186/s12984-025-01549-4 40325460 PMC12051271

[B13] Chow V. W. K. Ellmers T. J. Young W. R. Mak T. C. T. Wong T. W. L. (2018). Revisiting the relationship between internal focus and balance control in young and older adults. *Front. Neurol.* 9:1131. 10.3389/fneur.2018.01131 30687212 PMC6333651

[B14] Chu C. K. H. Wong T. W. L. (2019). Conscious postural control during standing on compliant surface by older adults. *J. Mot. Behav.* 51 342–350. 10.1080/00222895.2018.1481820 30081761

[B15] Degani A. M. Leonard C. T. Danna-Dos-Santos A. (2017). The effects of early stages of aging on postural sway: a multiple domain balance assessment using a force platform. *J. Biomech.* 64 8–15. 10.1016/j.jbiomech.2017.08.029 28893391

[B16] Eisma J. Rawls E. Long S. Mach R. Lamm C. (2021). Frontal midline theta differentiates separate cognitive control strategies while still generalizing the need for cognitive control. *Sci. Rep.* 11:14641. 10.1038/s41598-021-94162-z 34282209 PMC8290013

[B17] Ganz D. A. Latham N. K. (2020). Prevention of falls in community-dwelling older adults. *N. Engl. J. Med.* 382 734–743. 10.1056/NEJMcp1903252 32074420

[B18] George R. J. Hinder M. R. Puri R. Walker E. Callisaya M. L. (2021). Functional near-infrared spectroscopy reveals the compensatory potential of pre-frontal cortical activity for standing balance in young and older adults. *Neuroscience* 452 208–218. 10.1016/j.neuroscience.2020.10.027 33197501

[B19] Giovanni A. Capone F. di Biase L. Ferreri F. Florio L. Guerra A. et al. (2017). Oscillatory activities in neurological disorders of elderly: biomarkers to target for neuromodulation. *Front. Aging Neurosci.* 9:189. 10.3389/fnagi.2017.00189 28659788 PMC5468377

[B20] Gramann K. Gwin J. T. Ferris D. P. Oie K. Jung T. P. Lin C. T. et al. (2011). Cognition in action: imaging brain/body dynamics in mobile humans. *Rev. Neurosci.* 22 593–608. 10.1515/rns.2011.047 22070621

[B21] Herold F. Wiegel P. Scholkmann F. Müller N. G. (2018). Applications of functional near-infrared spectroscopy (fNIRS) neuroimaging in exercise cognition science: a systematic, methodology-focused review. *J. Clin. Med.* 7:466. 10.3390/jcm7120466 30469482 PMC6306799

[B22] Herold F. Wiegel P. Scholkmann F. Thiers A. Hamacher D. Schega L. (2017). Functional near-infrared spectroscopy in movement science: a systematic review on cortical activity in postural and walking tasks. *Neurophotonics* 4:041403. 10.1117/1.NPh.4.4.041403 28924563 PMC5538329

[B23] Hinderaker M. Sylcott B. Williams K. Lin C. C. (2020). Aging affects the ability to process the optic flow stimulations: a functional near-infrared spectrometry study. *J. Mot. Behav.* 52 466–473. 10.1080/00222895.2019.1645639 31361196

[B24] Holtzer R. Mahoney J. R. Izzetoglu M. Izzetoglu K. Onaral B. Verghese J. (2011). fNIRS study of walking and walking while talking in young and old individuals. *J. Gerontol. A Biol. Sci. Med. Sci.* 66 879–887. 10.1093/gerona/glr068 21593013 PMC3148759

[B25] Horak F. B. (2006). Postural orientation and equilibrium: What do we need to know about neural control of balance to prevent falls? *Age Ageing* 35(Suppl._2), ii7–ii11. 10.1093/ageing/afl077 16926210

[B26] Hu Y. Petruzzello S. J. Hernandez M. E. (2023). Beta cortical oscillatory activities and their relationship to postural control in a standing balance demanding test: influence of aging. *Front. Aging Neurosci.* 15:1126002. 10.3389/fnagi.2023.1126002 37213543 PMC10196243

[B27] Huang C. Y. Lin L. L. Hwang I. S. (2017). Age-related differences in reorganization of functional connectivity for a dual task with increasing postural destabilization. *Front. Aging Neurosci.* 9:96. 10.3389/fnagi.2017.00096 28446874 PMC5388754

[B28] Huang H. J. Ferris D. P. (2023). Non-invasive brain imaging to advance the understanding of human balance. *Curr. Opin. Biomed. Eng.* 28:100505. 10.1016/j.cobme.2023.100505 38250696 PMC10795750

[B29] Hülsdünker T. Mierau A. Neeb C. Kleinöder H. Strüder H. (2015). Cortical processes associated with continuous balance control as revealed by EEG spectral power. *Neurosci. Lett.* 592 1–5. 10.1016/j.neulet.2015.02.049 25724275

[B30] Jacobs J. V. Horak F. B. (2007). Cortical control of postural responses. *J. Neural Transm.* 114 1339–1348. 10.1007/s00702-007-0657-0 17393068 PMC4382099

[B31] Jasper H. H. (1958). Ten-twenty electrode system of the international federation. *Electroencephalogr. Clin. Neurophysiol.* 10 371–375. 10590970

[B32] Kahya M. Gouskova N. A. Lo O.-Y. Zhou J. Cappon D. Finnerty E. et al. (2022). Brain activity during dual-task standing in older adults. *J. NeuroEng. Rehabil.* 19:123. 10.1186/s12984-022-01095-3 36369027 PMC9652829

[B33] Kahya M. Li Y. Zhou J. Lo O. Y. Cappon D. B. Pascual-Leone A. et al. (2026). Characterization of EEG power spectrum correlates of standing and walking in older adults: a scoping review. *Exp. Gerontol.* 216:113075. 10.1016/j.exger.2026.113075 41720225

[B34] Kahya M. Moon S. Ranchet M. Vukas R. R. Lyons K. E. Pahwa R. et al. (2019). Brain activity during dual task gait and balance in aging and age-related neurodegenerative conditions: a systematic review. *Exp. Gerontol.* 128:110756. 10.1016/j.exger.2019.110756 31648005 PMC6876748

[B35] Kan C. Zhu S. Zhuang R. Wang Q. Geng A. Wang C. et al. (2025). Differences in cortical activation characteristics between younger and older adults during single/dual-tasks: a cross-sectional study based on fNIRS. *Biomed. Signal Process. Control* 99:106945. 10.1016/j.bspc.2024.106945

[B36] Klimesch W. (2012). α-band oscillations, attention, and controlled access to stored information. *Trends Cogn. Sci.* 16 606–617. 10.1016/j.tics.2012.10.007 23141428 PMC3507158

[B37] Kmet L. M. Cook L. S. Lee R. C. (2004). *Standard Quality Assessment Criteria for Evaluating Primary Research Papers from a Variety of Fields.* Edmonton, AB: Alberta Heritage Foundation for Medical Research.

[B38] Lehmann N. Kuhn Y. A. Keller M. Aye N. Herold F. Draganski B. et al. (2022). Brain activation during active balancing and its behavioral relevance in younger and older adults: a functional near-infrared spectroscopy (fNIRS) study. *Front. Aging Neurosci.* 14:828474. 10.3389/fnagi.2022.828474 35418854 PMC8997341

[B39] Lenz M. Tegenthoff M. Kohlhaas K. Stude P. Höffken O. Gatica Tossi M. A. et al. (2012). Increased excitability of somatosensory cortex in aged humans is associated with impaired tactile acuity. *J. Neurosci.* 32 1811–1816. 10.1523/jneurosci.2722-11.2012 22302820 PMC6703354

[B40] Li D. K. T. Mak T. C. T. Wong T. W. L. (2022). Does an externally focused dual-task mitigate real-time conscious postural control in older adults? *Exp. Aging Res.* 48 295–310. 10.1080/0361073x.2021.1982343 34590545

[B41] Li K. Z. Bherer L. Mirelman A. Maidan I. Hausdorff J. M. (2018). Cognitive involvement in balance, gait and dual-tasking in aging: a focused review from a neuroscience of aging perspective. *Front. Neurol.* 9:913. 10.3389/fneur.2018.00913 30425679 PMC6219267

[B42] Lin C. C. Barker J. W. Sparto P. J. Furman J. M. Huppert T. J. (2017). Functional near-infrared spectroscopy (fNIRS) brain imaging of multi-sensory integration during computerized dynamic posturography in middle-aged and older adults. *Exp. Brain Res.* 235 1247–1256. 10.1007/s00221-017-4893-8 28197672 PMC5494712

[B43] Lin C.-L. Hsieh Y.-W. Chen H.-Y. (2021). Age-related differences in alpha and beta band activity in sensory association brain areas during challenging sensory tasks. *Behav. Brain Res.* 408:113279. 10.1016/j.bbr.2021.113279 33812990

[B44] Lindsay S. Thiyagarajah K. (2021). The impact of service dogs on children, youth and their families: a systematic review. *Disabil. Health J.* 14:101012. 10.1016/j.dhjo.2020.101012 33069669

[B45] Mahoney J. R. Holtzer R. Verghese J. (2014). Visual-somatosensory integration and balance: evidence for psychophysical integrative differences in aging. *Multisensory Res.* 27 17–42. 10.1163/22134808-00002444 25102664 PMC4280078

[B46] Mahoney J. R. Li P. C. C. Oh-Park M. Verghese J. Holtzer R. (2011). Multisensory integration across the senses in young and old adults. *Brain Res.* 1426 43–53. 10.1016/j.brainres.2011.09.017 22024545 PMC3232048

[B47] Malcolm B. R. Foxe J. J. Joshi S. Verghese J. Mahoney J. R. Molholm S. et al. (2021). Aging-related changes in cortical mechanisms supporting postural control during base of support and optic flow manipulations. *Eur. J. Neurosci.* 54 8139–8157. 10.1111/ejn.15004 33047390

[B48] Marusic U. Taube W. Morrison S. A. Biasutti L. Grassi B. De Pauw K. et al. (2019). Aging effects on prefrontal cortex oxygenation in a posture-cognition dual-task: an fNIRS pilot study. *Eur. Rev. Aging Phys. Act* 16:2. 10.1186/s11556-018-0209-7 30655911 PMC6329111

[B49] Ozdemir R. A. Contreras-Vidal J. L. Lee B. C. Paloski W. H. (2016). Cortical activity modulations underlying age-related performance differences during posture-cognition dual tasking. *Exp. Brain Res.* 234 3321–3334. 10.1007/s00221-016-4730-5 27443853

[B50] Ozdemir R. A. Contreras-Vidal J. L. Paloski W. H. (2018). Cortical control of upright stance in elderly. *Mech. Ageing Dev.* 169 19–31. 10.1016/j.mad.2017.12.004 29277586

[B51] Page M. J. McKenzie J. E. Bossuyt P. M. Boutron I. Hoffmann T. C. Mulrow C. D. et al. (2021). The PRISMA 2020 statement: an updated guideline for reporting systematic reviews. *Bmj* 372:n71. 10.1136/bmj.n71 33782057 PMC8005924

[B52] Palmer J. A. Payne A. M. Ting L. H. Borich M. R. (2021). Cortical engagement metrics during reactive balance are associated with distinct aspects of balance behavior in older adults. *Front. Aging Neurosci.* 13:684743. 10.3389/fnagi.2021.684743 34335230 PMC8317134

[B53] Pan J. Tang H. (2025). Age-related effects on dynamic postural stability and prefrontal cortex activation during precision fitting tasks. *PeerJ* 13:e18548. 10.7717/peerj.18548 39897502 PMC11786713

[B54] Pan J. Zhang S. (2025). Dual-task effect on center of pressure oscillations and prefrontal cortex activation between young and older adults. *Res. Q. Exerc. Sport* 96 85–95. 10.1080/02701367.2024.2365940 38986156

[B55] Payne A. M. Palmer J. A. McKay J. L. Ting L. H. (2021). Lower cognitive set shifting ability is associated with stiffer balance recovery behavior and larger perturbation-evoked cortical responses in older adults. *Front. Aging Neurosci.* 13:742243. 10.3389/fnagi.2021.742243 34938171 PMC8685437

[B56] Peskar M. Manganotti P. Marusic U. Gramann K. (2025). Neurophysiological underpinnings of balance control and cognitive-motor interaction in early Parkinson’s disease. *Sci. Rep.* 15:25082. 10.1038/s41598-025-06777-1 40645984 PMC12254356

[B57] Peterson S. M. Ferris D. P. (2019). Group-level cortical and muscular connectivity during perturbations to walking and standing balance. *Neuroimage* 198 93–103. 10.1016/j.neuroimage.2019.05.038 31112786 PMC6592721

[B58] Petrigna L. Gentile A. Mani D. Pajaujiene S. Zanotto T. Thomas E. et al. (2021). Dual-task conditions on static postural control in older adults: a systematic review and meta-analysis. *J. Aging Phys. Act* 29 162–177. 10.1123/japa.2019-0474 32788414

[B59] Plichta M. M. Heinzel S. Ehlis A. C. Pauli P. Fallgatter A. J. (2007). Model-based analysis of rapid event-related functional near-infrared spectroscopy (NIRS) data: a parametric validation study. *Neuroimage* 35 625–634. 10.1016/j.neuroimage.2006.11.028 17258472

[B60] Pollock A. S. Durward B. R. Rowe P. J. Paul J. P. (2000). What is balance? *Clin. Rehabil.* 14 402–406. 10.1191/0269215500cr342oa 10945424

[B61] Reuter-Lorenz P. A. Cappell K. A. (2008). Neurocognitive aging and the compensation hypothesis. *Curr. Dir. Psychol. Sci.* 17 177–182. 10.1111/j.1467-8721.2008.00570.x

[B62] Rond V. Orcioli-Silva D. Dijkstra B. W. Orban de Xivry J. J. Pantall A. Nieuwboer A. (2021). Compromised brain activity with age during a game-like dynamic balance task: single- vs. dual-task performance. *Front. Aging Neurosci.* 13:657308. 10.3389/fnagi.2021.657308 34290599 PMC8287632

[B63] Rosso A. L. Cenciarini M. Sparto P. J. Loughlin P. J. Furman J. M. Huppert T. J. (2017). Neuroimaging of an attention demanding dual-task during dynamic postural control. *Gait Posture* 57 193–198. 10.1016/j.gaitpost.2017.06.013 28662465 PMC5585862

[B64] Rubega M. Formaggio E. Di Marco R. Bertuccelli M. Tortora S. Menegatti E. et al. (2021). Cortical correlates in upright dynamic and static balance in the elderly. *Sci. Rep.* 11:14132. 10.1038/s41598-021-93556-3 34238987 PMC8266885

[B65] Saadat Z. Sinaei E. Pirouzi S. Ghofrani M. Nami M. (2021). Cortical activity during postural recovery in response to predictable and unpredictable perturbations in healthy young and older adults: a quantitative EEG assessment. *Basic Clin. Neurosci.* 12 291–300. 10.32598/bcn.12.2.453.1 34925725 PMC8672669

[B66] Schoene D. Heller C. Aung Y. N. Sieber C. C. Kemmler W. Freiberger E. (2019). A systematic review on the influence of fear of falling on quality of life in older people: Is there a role for falls? *Clin. Interv. Aging* 14 701–719. 10.2147/cia.S197857 31190764 PMC6514257

[B67] Seeber M. Scherer R. Wagner J. Solis-Escalante T. Müller-Putz G. R. (2014). EEG beta suppression and low gamma modulation are different elements of human upright walking. *Front. Hum. Neurosci.* 8:485. 10.3389/fnhum.2014.00485 25071515 PMC4086296

[B68] Seidler R. D. Bernard J. A. Burutolu T. B. Fling B. W. Gordon M. T. Gwin J. T. et al. (2010). Motor control and aging: links to age-related brain structural, functional, and biochemical effects. *Neurosci. Biobehav. Rev.* 34 721–733. 10.1016/j.neubiorev.2009.10.005 19850077 PMC2838968

[B69] Strangman G. Boas D. A. Sutton J. P. (2002). Non-invasive neuroimaging using near-infrared light. *Biol. Psychiatry* 52 679–693. 10.1016/s0006-3223(02)01550-0 12372658

[B70] Teo W. P. Goodwill A. M. Hendy A. M. Muthalib M. Macpherson H. (2018). Sensory manipulation results in increased dorsolateral prefrontal cortex activation during static postural balance in sedentary older adults: an fNIRS study. *Brain Behav.* 8:e01109. 10.1002/brb3.1109 30230687 PMC6192391

[B71] Tsai Y. Y. Chen Y. C. Zhao C. G. Hwang I. S. (2022). Adaptations of postural sway dynamics and cortical response to unstable stance with stroboscopic vision in older adults. *Front. Physiol.* 13:919184. 10.3389/fphys.2022.919184 36105297 PMC9465385

[B72] Wang G. Yang Y. Liu X. Hua A. Luo X. Cai Y. et al. (2025). Neural connectivity and balance control in aging: insights from directed cortical networks during sensory conflict. *NeuroImage* 312:121218. 10.1016/j.neuroimage.2025.121218 40239853

[B73] Wang Z. Graci V. Seacrist T. Guez A. Keshner E. A. (2023). Localizing EEG recordings associated with a balance threat during unexpected postural translations in young and elderly adults. *IEEE Trans. Neural Syst. Rehabil. Eng.* 31 4514–4520. 10.1109/tnsre.2023.3331211 37938961 PMC10683785

[B74] Wittenberg E. Thompson J. Nam C. S. Franz J. R. (2017). Neuroimaging of human balance control: a systematic review. *Front. Hum. Neurosci.* 11:170. 10.3389/fnhum.2017.00170 28443007 PMC5385364

[B75] Xu G. Zhou M. Chen Y. Song Q. Sun W. Wang J. (2024a). Brain activation during standing balance control in dual-task paradigm and its correlation among older adults with mild cognitive impairment: a fNIRS study. *BMC Geriatr.* 24:144. 10.1186/s12877-024-04772-1 38341561 PMC10859010

[B76] Xu G. Zhou M. Wang J. Mao D. Sun W. (2024b). The effect of sensory manipulation on the static balance control and prefrontal cortex activation in older adults with mild cognitive impairment: a functional near-infrared spectroscopy (fNIRS) study. *BMC Geriatr.* 24:1020. 10.1186/s12877-024-05624-8 39702053 PMC11660590

[B77] Yu S. H. Hwang I. S. Huang C. Y. (2019). Neuronal responses to a postural dual-task with differential attentional prioritizations: compensatory resource allocation with healthy aging. *J. Gerontol. B Psychol. Sci. Soc. Sci.* 74 1326–1334. 10.1093/geronb/gby073 29955844

[B78] Zarahn E. Rakitin B. Abela D. Flynn J. Stern Y. (2007). Age-related changes in brain activation during a delayed item recognition task. *Neurobiol. Aging* 28 784–798. 10.1016/j.neurobiolaging.2006.03.002 16621168

