# Supplementary material for: Age-related cortical mechanisms underlying postural control: a systematic review
Source: Front Aging Neurosci. 2026 Sep 7;18:1879821. doi: 10.3389/fnagi.2026.1879821 (PMC13590551; doi:10.3389/fnagi.2026.1879821)
Supplement: Supplementary file 1 [file Data_Sheet_1.docx]

Supplementary Table 1. Search strategies

| MEDLINE | S1. exp Aging/  S2. ("older adult*" or "elderly" or "older person*" or "geriatric*").mp.  S3. S1 or S2  S4. exp Postural balance/  S5. ("Balance" OR "Postur*" OR "equilibrium" OR "sensory" OR "dual task*").mp  S6. S3 or S4  S7. exp Electroencephalography/ or exp Spectroscopy, Near-Infrared/  S8. ("fNIRS" OR "EEG" OR "neural activity" OR "cortical activit*" OR "functional connectivity" OR "event-related potential" OR "ERP" OR "oscillatory activity").mp  S9. S7 or S8  S10. S3 or S6 or S9 |
| --- | --- |
| EMBASE | ('aging'/exp OR 'older adult*' OR 'elderly' OR 'older person* or geriatric*') AND ('body equilibrium'/exp OR 'balance' OR 'postur*' OR 'equilibrium' OR 'sensory' OR 'dual task*') AND ('electroencephalogram'/exp OR 'near infrared spectroscopy'/exp OR 'fnirs' OR 'eeg' OR 'neural activity' OR 'cortical activit*' OR 'functional connectivity' OR 'event-related potential' OR 'erp' OR 'oscillatory activity') |
| CINAHL  PsycINFO | ((MH "Aging") OR ("older adult*" or "elderly" or "older person*" or "geriatric*")) AND ((MH "Balance, Postural") OR ("Balance" OR "Postur*" OR "equilibrium" OR "sensory" OR "dual task*")) AND ((MH "Electroencephalography" OR MH "Spectroscopy, Near-Infrared") OR ("fNIRS" OR "EEG" OR "neural activity" OR "cortical activit*" OR "functional connectivity" OR "event-related potential" OR "ERP" OR "oscillatory activity")) |

| Supplementary Table 2. Quality assessment of studies | | | | | | | | | | | | | | | | |
| --- | --- | --- | --- | --- | --- | --- | --- | --- | --- | --- | --- | --- | --- | --- | --- | --- |
| Study | C1 | C2 | C3 | C4 | C5 | C6 | C7 | C8 | C9 | C10 | C11 | C12 | C13 | C14 | Final | Quality |
| **EEG studies** | | | | | | | | | | | | | | | | |
| Bohle et al. 2019 | 2 | 2 | 1 | 2 | N/A | N/A | N/A | 2 | 1 | 2 | 0 | 1 | 2 | 2 | 0.77 | Good |
| Chan et al. 2019 | 2 | 2 | 2 | 2 | N/A | N/A | N/A | 2 | 1 | 2 | 0 | 2 | 1 | 2 | 0.82 | Strong |
| Chang et al. 2016 | 2 | 1 | 1 | 2 | N/A | N/A | N/A | 2 | 1 | 2 | 1 | 0 | 1 | 1 | 0.64 | Low |
| Chen et al.2021 | 2 | 2 | 1 | 2 | N/A | N/A | N/A | 2 | 2 | 2 | 2 | 0 | 2 | 2 | 0.82 | Strong |
| Chen et al.2024 | 2 | 2 | 1 | 1 | N/A | N/A | N/A | 2 | 2 | 2 | 2 | 1 | 2 | 2 | 0.86 | Strong |
| Chen et al.2025 | 1 | 2 | 1 | 1 | N/A | N/A | N/A | 2 | 1 | 2 | 1 | 1 | 2 | 2 | 0.73 | Good |
| Chow et al. 2018 | 2 | 2 | 2 | 2 | N/A | N/A | N/A | 2 | 1 | 2 | 1 | 0 | 1 | 2 | 0.77 | Good |
| Chu et al. 2019 | 2 | 2 | 2 | 2 | N/A | N/A | N/A | 2 | 1 | 1 | 2 | 0 | 1 | 1 | 0.73 | Good |
| Hu et al. 2023 | 2 | 2 | 2 | 2 | N/A | N/A | N/A | 2 | 1 | 2 | 2 | 1 | 2 | 2 | 0.91 | Strong |
| Huang et al. 2017 | 2 | 2 | 2 | 2 | N/A | N/A | N/A | 2 | 1 | 2 | 2 | 0 | 2 | 2 | 0.86 | Strong |
| Kahya et al. 2022 | 2 | 2 | 2 | 2 | N/A | N/A | N/A | 2 | 1 | 2 | 2 | 1 | 2 | 2 | 0.91 | Strong |
| Li et al. 2022 | 2 | 2 | 1 | 2 | N/A | N/A | N/A | 2 | 1 | 2 | 2 | 2 | 1 | 2 | 0.86 | Strong |
| Lin et al. 2021 | 2 | 2 | 2 | 2 | N/A | N/A | N/A | 2 | 1 | 2 | 2 | 1 | 2 | 2 | 0.91 | Strong |
| Malcolm et al. 2021 | 2 | 2 | 1 | 2 | N/A | N/A | N/A | 2 | 1 | 2 | 2 | 1 | 2 | 2 | 0.86 | Strong |
| Ozdemir et al. 2016 | 2 | 2 | 1 | 2 | N/A | N/A | N/A | 2 | 0 | 2 | 2 | 1 | 2 | 2 | 0.82 | Strong |
| Ozdemir et al. 2018 | 2 | 2 | 1 | 2 | N/A | N/A | N/A | 2 | 1 | 2 | 2 | 1 | 2 | 2 | 0.86 | Strong |
| Palmer et al. 2021 | 2 | 2 | 2 | 2 | N/A | N/A | N/A | 2 | 1 | 2 | 1 | 1 | 2 | 2 | 0.86 | Strong |
| Payne et al. 2021 | 2 | 2 | 2 | 2 | N/A | N/A | N/A | 2 | 1 | 2 | 1 | 1 | 2 | 2 | 0.86 | Strong |
| Rubega et al. 2021 | 2 | 2 | 1 | 1 | N/A | N/A | N/A | 2 | 1 | 1 | 2 | 1 | 2 | 2 | 0.77 | Good |
| Saadat et al. 2021 | 2 | 2 | 2 | 2 | N/A | N/A | N/A | 2 | 1 | 2 | 2 | 0 | 2 | 2 | 0.86 | Strong |
| Tsai et al. 2022 | 2 | 2 | 2 | 2 | N/A | N/A | N/A | 2 | 1 | 2 | 2 | 1 | 2 | 2 | 0.91 | Strong |
| Wang et al. 2025 | 2 | 2 | 2 | 1 | N/A | N/A | N/A | 2 | 1 | 2 | 2 | 1 | 2 | 2 | 0.86 | Strong |
| Wang et al. 2023 | 2 | 2 | 2 | 2 | N/A | N/A | N/A | 2 | 1 | 2 | 2 | 1 | 2 | 2 | 0.91 | Strong |
| Yu et al. 2019 | 2 | 2 | 1 | 1 | N/A | N/A | N/A | 2 | 2 | 2 | 2 | 1 | 2 | 2 | 0.86 | Strong |
| **fNIRS studies** | | | | | | | | | | | | | | | | |
| George et al. 2021 | 2 | 2 | 2 | 2 | N/A | N/A | N/A | 2 | 1 | 2 | 1 | 1 | 2 | 2 | 0.86 | Strong |
| Hinderaker et al. 2020 | 2 | 2 | 1 | 2 | N/A | N/A | N/A | 2 | 1 | 2 | 1 | 1 | 2 | 2 | 0.82 | Strong |
| Kan et al. 2025 | 2 | 2 | 2 | 2 | N/A | N/A | N/A | 2 | 2 | 1 | 1 | 1 | 2 | 2 | 0.86 | Strong |
| Lehmann et al. 2022 | 2 | 2 | 2 | 2 | N/A | N/A | N/A | 2 | 2 | 2 | 2 | 2 | 2 | 2 | 1.00 | Strong |
| Lin et al. 2017 | 1 | 2 | 1 | 2 | N/A | N/A | N/A | 2 | 1 | 2 | 1 | 1 | 2 | 2 | 0.77 | Good |
| Marusic et al. 2019 | 2 | 2 | 2 | 2 | N/A | N/A | N/A | 2 | 0 | 2 | 2 | 1 | 2 | 2 | 0.86 | Strong |
| Pan et al. 2025 | 2 | 2 | 0 | 1 | N/A | N/A | N/A | 2 | 2 | 2 | 2 | 1 | 2 | 2 | 0.82 | Strong |
| Pan et al. 2025 | 2 | 2 | 1 | 1 | N/A | N/A | N/A | 2 | 2 | 2 | 2 | 1 | 2 | 2 | 0.86 | Strong |
| Rond et al. 2021 | 2 | 2 | 2 | 2 | N/A | N/A | N/A | 2 | 1 | 2 | 2 | 1 | 2 | 2 | 0.91 | Strong |
| Rosso et al. 2017 | 1 | 2 | 2 | 2 | N/A | N/A | N/A | 2 | 1 | 2 | 0 | 1 | 2 | 2 | 0.77 | Good |
| Teo et al. 2018 | 2 | 2 | 0 | 2 | N/A | N/A | N/A | 2 | 1 | 2 | 2 | 1 | 2 | 2 | 0.82 | Strong |
| Xu et al. 2024 | 2 | 2 | 2 | 2 | N/A | N/A | N/A | 2 | 2 | 2 | 2 | 1 | 2 | 2 | 0.95 | Strong |
| Xu et al. 2024 | 2 | 2 | 1 | 2 | N/A | N/A | N/A | 2 | 2 | 2 | 1 | 1 | 2 | 2 | 0.86 | Strong |
| **# of “0” scores** | 0 | 0 | 2 | 0 | N/A | N/A | N/A | 0 | 2 | 0 | 3 | 6 | 0 | 0 |  |  |
| **# of “1” scores** | 3 | 1 | 15 | 7 | N/A | N/A | N/A | 0 | 26 | 3 | 10 | 28 | 5 | 2 |  |  |
| *Note*. C: Criteria; Yes=2, Partial=1, No=0, N/A: Not applicable; Final score is indicative of low quality (< 0.7), good quality (0.7-0.79), and strong quality (≥ 0.8).  C1: Question / objective sufficiently described?  C2: Study design evident and appropriate?  C3: Method of subject/comparison group selection or source of information/input variables described and appropriate?  C4: Subject (and comparison group, if applicable) characteristics sufficiently described?  C5: If interventional and random allocation was possible, was it described?  C6: If interventional and blinding of investigators was possible, was it reported?  C7: If interventional and blinding of subjects was possible, was it reported?  C8: Outcome and (if applicable) exposure measure(s) well defined and robust to measurement / misclassification bias? Means of assessment reported?  C9: Sample size appropriate?  C10: Analytic methods described/justified and appropriate?  C11: Some estimate of variance is reported for the main results?  C12: Controlled for confounding?  C13: Results reported in sufficient detail?  C14: Conclusions supported by the results? | | | | | | | | | | | | | | | | |
